# AAT_Rhg1_ is a tonoplast protein that alters amino acid, metabolic, and defense responses and nematode resistance

**DOI:** 10.1093/plphys/kiag706

**Published:** 2026-09-21

**Authors:** Yulin Du, Aaron Lowenstein, Jorge El-Azaz, Hiroshi A Maeda, Andrew F Bent

**Affiliations:** Department of Plant Pathology, University of Wisconsin—Madison, 1630 Linden Drive, Madison, WI 53706, United States; Department of Plant Pathology, University of Wisconsin—Madison, 1630 Linden Drive, Madison, WI 53706, United States; Department of Botany, University of Wisconsin—Madison, 430 Lincoln Drive, Madison, WI 53706, United States; Department of Botany, University of Wisconsin—Madison, 430 Lincoln Drive, Madison, WI 53706, United States; Cellular & Molecular Biology Program, University of Wisconsin—Madison, 1525 Linden Drive, Madison, WI 53706, United States; Department of Plant Pathology, University of Wisconsin—Madison, 1630 Linden Drive, Madison, WI 53706, United States

## Abstract

Soybean cyst nematode (SCN, *Heterodera glycines* [HG]) causes significant soybean yield losses. The *Rhg1* locus is a major contributor to SCN resistance and contains 3 genes that mediate this trait, including *Rhg1-GmAAT* (*Glyma.18G022400*), which encodes the putative amino acid transporter AAT_Rhg1_. The molecular function of AAT_Rhg1_ in SCN resistance is not understood. In this study, *rhg1-b* soybean lines with *Rhg1-GmAAT* silencing demonstrated that *Rhg1-GmAAT* can contribute to resistance against HG 0 SCN and also against problematic HG 2.5.7 populations that partially overcome *rhg1-b*-mediated resistance. When 2 previously characterized AAT_Rhg1_ mutants with contrasting effects on amino acid and betalain accumulation were tested for SCN resistance, the AAT_Rhg1_ Y268L mutant complemented SCN resistance in *Rhg1-GmAAT*-silenced plants, while the D122A mutant did not. Overexpression of *Rhg1-GmAAT* was not sufficient to enhance SCN resistance, suggesting that AAT_Rhg1_ requires coordinated activity with other proteins or pathways. Confocal microscopy demonstrated that AAT_Rhg1_ localizes to the tonoplast in soybean root cells. Amino acid, transcriptomic, and metabolomic profiles were determined for root segments collected 3 d after mock or SCN inoculation. In *Rhg1-GmAAT*-silenced *rhg1-b* plants relative to fully resistant (non-silenced) *rhg1-b* plants, levels of leucine, isoleucine, and tyrosine were significantly elevated. *Rhg1-GmAAT* silencing reduced SCN-responsive transcript abundances for multiple processes, significantly including genes for mitogen-activated protein kinase (MAPK) signaling, ethylene-associated responses, and starch and sucrose metabolism. The most common identified metabolomic changes were in amino acid derivatives, shikimate/phenylpropanoid/isoflavonoid compounds, terpenoids, and especially fatty acids. These findings can guide further investigation into the mechanisms by which AAT_Rhg1_ contributes to SCN resistance.

## Introduction

Soybean cyst nematode (SCN, *Heterodera glycines*) is responsible for substantial yield losses in major soybean production regions (Bandara et al. 2020; Bradley et al. 2021; Peng et al. 2021; Tylka and Marett 2021; Júnior et al. 2022; Barros et al. 2023). During the SCN life cycle, it establishes a multi-week biotrophic parasitism within soybean roots, and plant resistance may act by directly targeting the nematode or by disrupting the biotrophic interaction. Infective second-stage juveniles (J2) penetrate soybean roots and migrate to the periphery of the vascular cylinder, where they induce extensive host cell reprogramming to establish a specialized multinucleate, cytoplasm-rich feeding structure termed a syncytium (Kim et al. 2012; Zhang et al. 2017; Juvale and Baum 2018). Fertilized females develop eggs during parasitism and subsequently form cysts that encapsulate eggs, which can remain viable in soil for several years (Niblack et al. 2006).

Individual SCN populations are genetically diverse and can gradually overcome host resistance when soybean cultivars with similar SCN resistance are planted for multiple seasons (Gardner et al. 2017; McCarville et al. 2017; Hua et al. 2018; Meinhardt et al. 2021; Critchfield et al. 2023; Kleczewski et al. 2023; Chen and Potter 2026). SCN populations can be classified into *Heterodera glycines* types (HG types) based on their ability to reproduce on defined soybean indicator lines that carry distinct genetic resistance sources (Niblack et al. 2002). For example, HG 0 populations are not capable of overcoming any of the resistance indicator lines, whereas more virulent populations such as HG 2.5.7 can partially overcome “PI 88788”-type *rhg1-b* resistance, and HG 1.3.6.7 can partially overcome “Peking”-type resistance (Niblack et al. 2002). SCN populations with the same HG type can differ genetically and, even within a population, individual nematodes may have distinct mechanisms to overcome resistance.

Resistance to *Heterodera glycines* 1 (*Rhg1)* is the most extensively utilized SCN resistance QTL in soybean production (Concibido et al. 2004; Kim et al. 2012; Mitchum 2016; McCarville et al. 2017; Bent 2022). *Rhg1* genes reside on a 31 to 36 kb 4-gene block that exhibits DNA sequence polymorphisms and copy number variation (Cook et al. 2012, 2014). There are 3 predominant *Rhg1* haplotypes. *rhg1-a* (“Peking”-type) haplotypes often carry 3 tandem copies of the 4-gene block, *rhg1-b* (“PI 88788”-type) haplotypes typically contain 9 or 10 copies, while most SCN-susceptible soybean varieties such as Williams 82 (Wm82) carry 1 copy of the *rhg1-c* haplotype (Cook et al. 2012, 2014; Lee et al. 2015; Liu et al. 2017; Patil et al. 2019). The basal expression level of *Rhg1* genes positively correlates with *Rhg1* copy number (Cook et al. 2014).

Three genes within the *Rhg1* locus have been demonstrated to contribute to SCN resistance including *Glyma.18G022400* (*Rhg1-GmAAT*), which encodes the putative amino acid transporter AAT_Rhg1_, *Glyma.18G022500* (*GmSNAP18*) encoding alpha-soluble N-ethylmaleimide-sensitive factor attachment protein (α-SNAP_Rhg1_), and *Glyma.18G022700*, which encodes a poorly characterized protein with a wound-inducible domain (WI12_Rhg1_) (Cook et al. 2012; Liu et al. 2017; Dong and Hudson 2022). Resistant isoforms of α-SNAP_Rhg1_ are apparently toxic to syncytia (Bayless et al. 2016), but further molecular mechanisms by which the 3 Rhg1 proteins mediate SCN resistance are not well understood (Bent 2022).

AAT_Rhg1_, the focus of the present study, is predicted to belong to the tryptophan/tyrosine transporter protein family and contains 11 transmembrane domains (InterPro IPR013059, Pfam PF03222) (Paysan-Lafosse et al. 2023). AAT_Rhg1_ amino acid transport activity has not been demonstrated, but indirect evidence suggests a role of AAT_Rhg1_ in modulating amino acid levels in soybean roots. Overexpression of *Rhg1-GmAAT* altered root amino acid levels, systemic glutamate transport, and tolerance to exogenous glutamic acid (Guo et al. 2019). The conserved-site mutants D122A and Y268L of AAT_Rhg1_ had opposite impacts from each other on amino acid accumulation and betalain production (Du et al. 2025). At the cellular level, AAT_Rhg1_ accumulated on macrovesicle-like membrane structures in SCN-penetrated cells, and overexpression of *Rhg1-GmAAT* in soybean roots influenced membrane dynamics even in the absence of SCN infestation (Han et al. 2023). A recent study reported that *Rhg1-GmAAT* expression can be regulated by an AP2/ERF transcription factor, GmTINY (He et al. 2025). Together, these findings highlight a multifaceted role of AAT_Rhg1_ in metabolic and cellular responses. There is limited precedent for other plant amino acid transporters impacting nematode parasitism and plant disease resistance (Elashry et al. 2013; Jiang et al. 2022; Dutta et al. 2024; Yao et al. 2025).

How AAT_Rhg1_ affects SCN-responsive transcriptional and metabolic changes remains unclear. Previous transcriptomic studies have explored gene expression responses in soybean roots during SCN infection for various soybean genotypes, SCN HG types, sampled tissues, and time points after inoculation (eg Ithal et al. 2007a, 2007b; Klink et al. 2010; Kandoth et al. 2011; Song et al. 2019; Wei et al. 2022; Torabi et al. 2023; Sultana et al. 2024). Transcriptomic data have been integrated with metabolomic analyses to identify altered metabolic pathways associated with susceptibility or resistance to an HG 1.2.3.5.7 SCN population (Shi et al. 2021). However, these studies were not designed to dissect the role of *Rhg1-GmAAT*. The present study aimed to further characterize the contribution of AAT_Rhg1_ to SCN resistance and to gain information regarding underlying mechanisms.

## Materials and methods

More detailed descriptions of all utilized methods can be found in the Supplementary Information. A list of the oligonucleotide primers used is provided in Table S1.

### Plasmid constructs

The *Rhg1-GmAAT* silencing construct driven by a *GmUbi* promoter reported by Hernandez-Garcia et al. (2010) was described previously (Du et al. 2025). A synthetic artificial RNAi-escaping *Rhg1-GmAAT* coding sequence driven by a *GmUbi* promoter was created by designing synonymous codon substitutions with preference for commonly used soybean codons, synthesized at Twist Bioscience (South San Francisco, CA, USA) and deployed in binary vector pAGM4673, and also modified by site-directed mutagenesis to generate analogous *SynAAT_Rhg1_Y268L* or *SynAAT_Rhg1_D122A* constructs (Supplementary Information). Two different pAGM4673 constructs expressed wild-type (WT) AAT_Rhg1_, one native (genomic sequence spanning from 1,546 bp upstream of the 5′UTR start to 440 bp downstream of the 3′UTR terminus of *Rhg1-GmAAT*), and the other for constitutive overexpression (*Rhg1-GmAAT* coding sequence (exons only) controlled by a double CaMV *35S* promoter with a tobacco mosaic virus (TMV) *omega* enhancer, followed by the *nos* terminator).

For microscopy, genes encoding the fluorescent proteins mCherry, TdTomato with a C-terminal Lys-Asp-Glu-Leu (KDEL) endoplasmic reticulum (ER) retention signal, and mWasabi were obtained as Golden Gate level 0 plasmids. Subsequent cloning was done using the Golden Gate Assembly MoClo Tool Kit (Weber et al. 2011). Green fluorescent protein mWasabi was tagged to the N- or C-terminus of AAT_Rhg1_ in Golden Gate level 0 to level 1 reactions. The loop-tagged mWasabi AAT_Rhg1_ was created through Gibson Assembly of PCR products. Vesicle-associated membrane protein 711 (VAMP711) was cloned from Arabidopsis Biological Resource Center (ABRC) CD3-781601, and inserted into the Golden Gate level 0 vector through BbsI overhangs added by PCR. The above genes were cloned into Golden Gate level 1 vectors for expression under the control of the double CaMV *35S* promoter with TMV *omega* translational enhancer and *nos* terminator. The level 1 modules were assembled into the Golden Gate level 2 binary vector to generate constructs for co-expression in soybean roots.

### Transgenic plant materials

Whole transgenic *Rhg1-GmAAT*-silenced soybean plants were generated from IL3025N (*rhg1-b* SCN resistance haplotype) at Wisconsin Crop Innovation Center (WCIC) as described previously and in Supplemental Information (Du et al. 2025). Transgene expression or target gene silencing was verified by reverse transcription polymerase chain reaction (RT-qPCR); primer sequences are provided in Table S1. For the *Rhg1-GmAAT*-silenced RNAi and corresponding empty vector (EV) lines, stable homozygous transgenic lines were selected, and fourth-generation (T4) transgenic plants were used in SCN assays. *Rhg1-GmAAT* overexpression soybean lines were generated at WCIC. For overexpression in the Wm82 background, homozygous T2 plants were used in SCN assays. For overexpression in IL3025N (*rhg1-b*) and IL3849N (*rhg1-a* + *Rhg4* SCN resistance) (Haarith et al. 2026), T1 plants were used in SCN assays. T-DNA copy number was tested for each T1 plant used in SCN assays to identify homozygotes, hemizygotes, and non-transgenic segregants.

Composite soybean plants with transgenic roots were generated from soybean seedling hypocotyls using *Agrobacterium rhizogenes* strain K599 following the methods of Estrada-Navarrete et al. (2007) and Fan et al. (2020). Detached transgenic soybean roots were produced from soybean cotyledons using the method described by Cook et al. (2012).

Homozygous transgenic *Arabidopsis thaliana* Col-0 lines expressing *Rhg1-GmAAT* were generated using the floral dip method (Clough and Bent 1998). *AtAVT6C* knockout line CS864997 was ordered from ABRC.

### DNA extraction and copy number

DNA was extracted from young soybean leaf tissue using the CTAB/chloroform method. Transgene copy number was tested by qPCR, measuring the relative level of the spectinomycin resistance gene (Martinell et al. 2013) on T-DNA relative to *Glyma.18G022800*, which is known to have only 1 copy per haploid genome.

### Confocal microscopy

Confocal images were acquired using a Zeiss LSM 980 with 40× water-immersion objective lens. TdTomato or mCherry signal was acquired with 561 nm laser excitation and 573 to 628 nm emission, while mWasabi signal was acquired with 488 nm laser excitation and 500 to 553 nm emission with beam splitter MBS 488/561 and laser intensity of 2.0%.

### SCN propagation and resistance assay

SCN populations were initially collected in the field and propagated in the laboratory on soybean plants grown in a 1:1 soil–sand mixture. The HG 0 population used in experiments was originally collected in Illinois. Two HG 2.5.7 populations were sourced from Minnesota (HG 2.5.7 MN) or Missouri (HG 2.5.7 MO). To maintain or enhance virulence, these SCN populations were propagated on different soybean varieties: HG 0 on the susceptible variety Wm82 and HG 2.5.7 on the *rhg1-b* variety IL3025N. Propagation pots were grown for approximately 2 mo before harvesting. SCN cysts and eggs were extracted using the method described by Cook et al. (2012). Eggs were hatched in 4 mM pH 5.6 ZnCl_2_ solution.

SCN resistance assays utilized anonymized individual plants in 6 cm pots containing a 1:1 soil–sand mixture, each inoculated with ∼1,000 SCN eggs and incubated for 35 d, while composite plants were each inoculated with ∼750 freshly hatched SCN J2s and incubated for 30 d, prior to cyst extraction and counting. Sugar beet cyst nematode (*Heterodera schachtii*, BCN) assays on Arabidopsis were conducted as described by Butler et al. (2019).

### RNA extraction, RT-qPCR, and RNA-seq

RNA from flash-frozen root samples was extracted using the Direct-zol RNA MiniPrep Plus Kit (Zymo Research, Irvine, CA, USA; see Supplementary Information). cDNA was synthesized using FIREScript RT cDNA synthesis MIX from Solis BioDyne (Tartu, Estonia). Quantitative RT-qPCR was performed using HOT FIREPol EvaGreen qPCR Supermix from Solis BioDyne and CFX96 real-time PCR detection system (Bio-Rad, Hercules, CA, USA).

For RNA-seq studies (see also Supplementary Information), 10-day-old soybean plants grown in sand were inoculated with 1,000 freshly hatched SCN (HG 0) J2s or the same volume of SCN-free hatching solution. Root segments with visible lesions caused by nematode penetration (or analogous regions from mock-inoculated plants) were flash-frozen within 1 min of root collection. Four replicate samples were analyzed for each genotype-treatment. Each replicate comprised multiple root sections from one individual plant. RNA-seq and data analysis were performed by Novogene (Sacramento, CA, USA) using their Eukaryotic mRNA-seq service (Illumina NovaSeq platform, 150 bp paired-end reads, ≥20 million read pairs per sample, read quality control, and gene expression quantification). The iAAT_Mock group had 3 replicates after 1 outlier was removed. Differential expression profiling primarily utilized the Novogene platform for fragments per kilobase of transcript per million mapped reads (FPKM), log2 fold change, and adjusted *P*-value (*P*_adj_) data, as well as Kyoto Encyclopedia of Genes and Genomes (KEGG) and Gene Ontology (GO) term functional group enrichment analysis.

### Metabolite extraction and quantification

For metabolite extraction (detailed in Supplementary Information), 12-day-old soybean plants grown in sand were inoculated with SCN (HG 0) and at 3 dpi root sections were harvested, as described above. For each sample, root sections from 3 individual plants were pooled, flash-frozen in liquid nitrogen, and freeze-dried for 48 h. Metabolites were extracted using a 2:1 mix of methanol and chloroform, with 50 μM of ^13^C_6_-ring-labeled-L-phenylalanine as internal standard. Liquid chromatography-mass spectrometry (LC-MS) analysis used a Vanquish Horizon Binary UHPLC (Thermo Scientific, Waltham, MA, USA) coupled to a Q Exactive Orbitrap mass spectrometer (Thermo Scientific, Waltham, MA, USA). Amino acids were analyzed by zwitterionic hydrophilic interaction liquid chromatography (HILIC-Z) using an InfinityLab Poroshell 120 HILIC-Z column. MS/MS settings for HILIC-Z chromatography were described by El-Azaz and Maeda (2024). Phenylpropanoids and other semi-polar metabolites were determined by reversed-phase (RP) chromatography using an Acquity UPLC HSS T3 column, in the positive ionization mode or the negative ionization mode. Untargeted metabolite data processing and metabolite annotation were performed using MZmine v4.0 (Schmid et al. 2023) and SIRIUS v5 (Dührkop et al. 2019) as described by El-Azaz and Maeda (2024). For targeted metabolite analysis, compound abundance was calculated based on the peak area of authentic standards and normalized to sample mass and internal standard recovery for each sample.

### Association analysis of transcriptome and metabolome data

To explore associations between transcriptomic and metabolomic responses to SCN, RNA-seq KEGG enrichment results were compared with compound-level metabolite KEGG enrichment results. Metabolite features with *P* < 0.05 from two-sided Welch's t-tests between SCN-treated and mock-treated roots within each genotype were mapped to KEGG compound IDs, deduplicated to unique compounds, and tested for pathway enrichment using a hypergeometric test. RNA-seq pathway significance was defined as *P_adj_* < 0.05, whereas metabolite pathway significance was considered exploratory and defined as raw *P* < 0.05. Biomni (https://biomni.stanford.edu) (Huang et al. 2026) was used for initial workflow exploration, and the final analyses and figures were independently reproduced and verified by the authors.

### Statistical analyses

Statistical analyses (see Supplementary Information) other than RNA-seq analyses were performed as described using R (version 4.4.2).

## Results

### *Rhg1-GmAAT* contributes to resistance against HG 0 and HG 2.5.7 SCN

Soybean resistance against SCN is quantitative (Bent 2022). To test the contribution of AAT_Rhg1_ to SCN resistance in intact soybean plants and against key SCN types, we generated *Rhg1-GmAAT*-silenced transgenic soybean lines using an RNAi construct in the high-copy *rhg1-b* soybean variety IL3025N. Silencing of *Rhg1-GmAAT* was verified by RT-qPCR, with transcript levels reduced to 20% to 40% of the control (Figure S1). Wm82, which lacks *rhg1-b*, was used as the SCN-susceptible control to calculate the within-experiment female index (FI = 100 for Wm82), which is the most widely utilized measure of SCN resistance (Niblack et al. 2002). Two independent EV transgenic lines and two independent *Rhg1-GmAAT* RNAi (iAAT) lines were tested against 3 SCN populations representing 2 HG types. We first utilized an SCN HG 0 population originally collected in Illinois, against which *rhg1-b* remains effective. HG 0 SCN are virulent on soybeans lacking *Rhg1* resistance (eg Wm82). As expected, the HG 0 SCN population exhibited a substantially lower female index on *rhg1-b* EV lines than on Wm82 (Fig. 1a). In the *rhg1-b* background, silencing *Rhg1-GmAAT* significantly increased the mean female index of HG 0 SCN relative to the same HG 0 SCN on EV lines, indicating compromised resistance (Fig. 1a). This result is consistent with previous silencing experiments conducted on detached transgenic roots (Cook et al. 2012), and further supports a role for *Rhg1-GmAAT* in SCN resistance.

**Figure 1:**
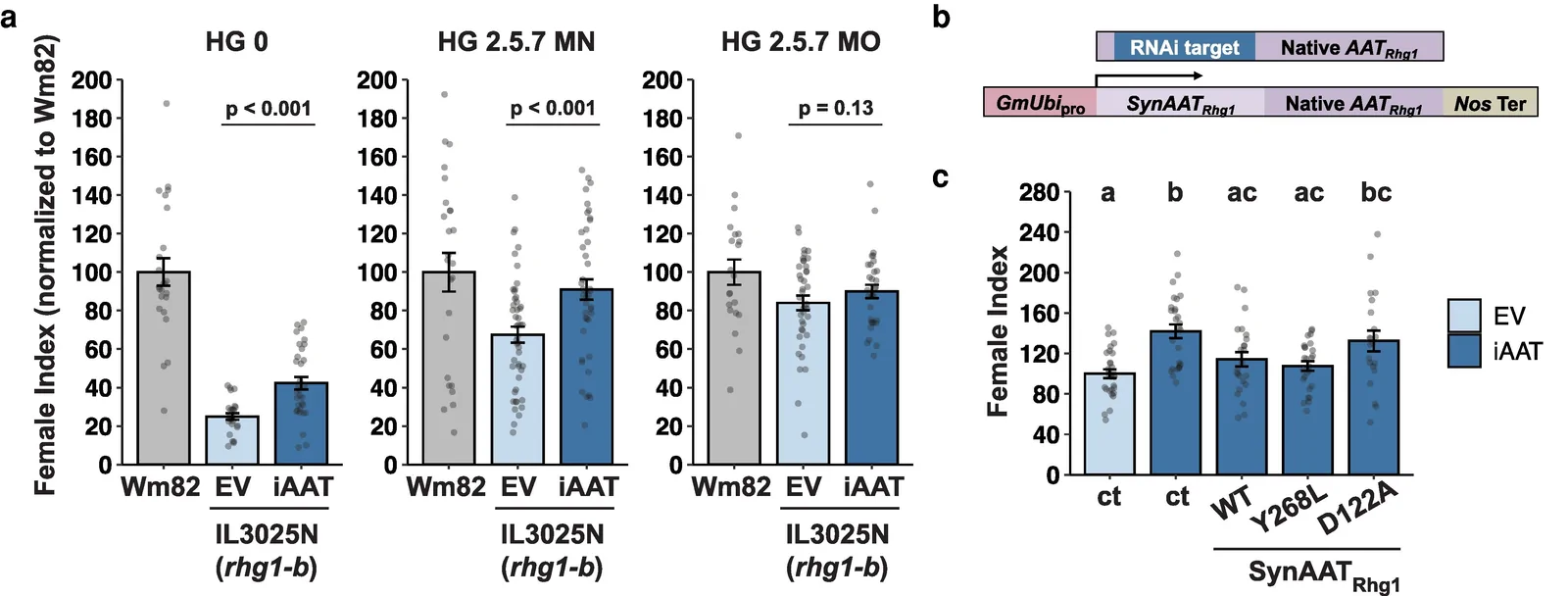
Silencing *Rhg1-GmAAT* in an *rhg1-b* background compromises SCN resistance. a) SCN resistance tests with Wm82 and transgenic lines, including 2 independent events each of IL3025N (*rhg1-b*) transformed with an EV or an *Rhg1-GmAAT* RNAi silencing construct (iAAT). SCN populations tested included HG 0, HG 2.5.7 from Minnesota (HG 2.5.7 MN), and Missouri (HG 2.5.7 MO). SCN resistance was measured using the FI = 100 × (number of cysts on a tested line/number of cysts on the susceptible control Wm82). Lower FI indicates stronger resistance. Each graph shows combined data from 2 independent events per transgenic genotype across 2 independent experiments. One-tailed *t*-tests were used to analyze the HG 2.5.7 MN and HG 2.5.7 MO experiments; the Wilcoxon rank-sum test was used for HG 0 due to non-normal distribution. Data are presented as mean ± SE. Each dot represents data from an individual plant (Wm82, *n* ≥ 22; EV or iAAT, *n* ≥ 34). b) Schematic of synthesized *Rhg1-GmAAT* (*SynAAT_Rhg1_*). An artificial, codon-substituted DNA fragment designed to escape the existing RNAi transgene was fused to the remaining native coding sequence to encode full-length AAT_Rhg1_. Expression was controlled by a soybean ubiquitin promoter (*GmUbi*_pro_) and a Nos terminator (*Nos* Ter). c) Functional complementation of SCN (HG 0) resistance in composite iAAT plants expressing *SynAAT_Rhg1_*. *SynAAT_Rhg1_* (WT, Y268L, or D122A) constructs were expressed in transgenic roots generated on iAAT plants. EV and iAAT roots expressing the GFP marker alone were used as controls (ct; EV-ct, resistant control; iAAT-ct, susceptible control). Female index was calculated relative to EV-ct (IL3025N). Data from 2 independent experiments were combined for analysis. No significant experiment effect was detected by two-way ANOVA. Bars sharing the same letter are not significantly different (Tukey's HSD post hoc test). Data are presented as mean ± SE. Each dot represents data from an individual plant (*n* ≥ 20).

Two HG 2.5.7 SCN populations originating from Minnesota (HG 2.5.7 MN) and Missouri (HG 2.5.7 MO), each partially overcoming *rhg1-b*-mediated SCN resistance, were then tested on *Rhg1-GmAAT*-silenced lines. On EV *rhg1-b* lines, both HG 2.5.7 populations showed higher female indices than HG 0, consistent with reduced effectiveness of high-copy *rhg1-b* against HG 2.5.7 SCN (Fig. 1a). Despite the elevated virulence of HG 2.5.7 MN on *rhg1-b*, silencing *Rhg1-GmAAT* resulted in significantly increased susceptibility compared with EV lines (Fig. 1a), indicating that *Rhg1-GmAAT* still contributes substantially to resistance against HG 2.5.7 MN. The HG 2.5.7 MO population showed a similar trend, although not statistically significant (*t*-test *P* = 0.13; Fig. 1a). The mean female index of HG 2.5.7 MO on *rhg1-b* EV lines was significantly higher than HG 2.5.7 MN (*t*-test *P* < 0.05; Fig. 1a), indicating that HG 2.5.7 MO is more virulent than HG 2.5.7 MN on a full *rhg1-b* background, even though both are classified as HG 2.5.7 populations.

A transgenic complementation test further validated the role of *Rhg1-GmAAT* in SCN resistance. We generated an artificial codon-substituted, RNAi-escaping *Rhg1-GmAAT* (*SynAAT_Rhg1_WT*; Fig. 1b). *SynAAT_Rhg1_WT* encodes a functional protein, as it enhanced betalain accumulation when co-overexpressed with *RUBY*, consistent with a prior study (He et al. 2020; Du et al. 2025) (Figure S2). For the complementation test, *SynAAT_Rhg1_WT* was overexpressed via a soybean ubiquitin promoter in transgenic roots of composite soybean plants derived from the *Rhg1-GmAAT*-silenced (iAAT) lines described above. When inoculated with SCN HG 0, overexpression of *SynAAT_Rhg1_WT* in the iAAT background restored resistance to levels equivalent to *rhg1-b* EV plants (EV-ct; Fig. 1c), indicating that the compromised resistance in the RNAi lines was successfully complemented.

### Functional consequences of AAT_Rhg1_ D122A and Y268L mutations in SCN resistance

In a recent study, we showed that soybean AAT_Rhg1_ Y268L and D122A mutations, at residues that are highly conserved across plant amino acid transporters, differentially affect AAT_Rhg1_ function in regulating amino acid homeostasis and *RUBY*-mediated betalain synthesis (Du et al. 2025). The D122A mutation apparently blocked AAT_Rhg1_ function, while the AAT_Rhg1_Y268L mutant was postulated to have increased activity as it induced a stronger betalain accumulation than wild-type AAT_Rhg1_ (Du et al. 2025). To examine whether these 2 mutants also affect AAT_Rhg1_-mediated SCN resistance, *SynAAT_Rhg1_Y268L* or *SynAAT_Rhg1_D122A* variants were overexpressed in *Rhg1-GmAAT* RNAi lines and tested against SCN HG 0. In transgenic roots, synthesized and/or native *Rhg1-GmAAT* transcript levels were measured by RT-qPCR (Figure S3). The Y268L mutant restored the compromised SCN resistance phenotype in the iAAT background, but did not confer greater resistance than *SynAAT_Rhg1_WT* (Fig. 1c). As predicted for a loss-of-function mutation, the D122A mutant did not differ significantly from iAAT control (iAAT-ct) plants, indicating that D122A failed to complement SCN resistance (Fig. 1c).

### Overexpressing *Rhg1-GmAAT* alone did not enhance SCN resistance

We previously reported that *rhg1-a*, *rhg1-b*, or *rhg1-c* encode identical AAT_Rhg1_ proteins, and that increased *Rhg1* copy number correlates with elevated *Rhg1-GmAAT* transcript levels and enhanced SCN resistance (Cook et al. 2014). In resistant soybean varieties, AAT_Rhg1_ protein abundance is further induced upon SCN infestation and accumulates along the SCN penetration path (Han et al. 2023). In the present study, we transgenically expressed *Rhg1-GmAAT* in various soybean genetic backgrounds to test whether constitutively elevated *Rhg1-GmAAT* expression enhances SCN resistance. A *35S promoter:Rhg1-GmAAT CDS:Nos terminator* construct was transformed into SCN-susceptible single-copy *rhg1-c* Wm82 and SCN-resistant low-copy *rhg1-a* variety IL3849N. A *native promoter:native Rhg1-GmAAT (UTR and introns included):native terminator* construct was transformed into Wm82 and SCN-resistant high-copy *rhg1-b* variety IL3025N. *Rhg1-GmAAT* transcript levels in independent transgenic lines were quantified by RT-qPCR (Figure S4). Transgene presence significantly elevated *Rhg1-GmAAT* transcript levels in most lines, but with the native construct, two lines (Wm82 Line 2 and *rhg1-b* Line 2) showed only marginal increases, and one line (*rhg1-b* Line 1) showed no significant change (Figure S4). Transgenic lines were then tested against SCN populations chosen for substantial virulence on the corresponding *rhg1* genetic backgrounds. Accordingly, Wm82 lines were tested with HG 0, IL3025N (*rhg1-b*) lines with HG 2.5.7, and IL3849N (*rhg1-a*/*Rhg4*) lines with HG 1.3.6.7. Elevation of *Rhg1-GmAAT* transcript levels by the above methods did not result in significantly enhanced SCN resistance relative to non-transformed plants or non-transgenic segregant controls (NT_seg_) in any tested genotype (Fig. 2). These results indicate that increasing *Rhg1-GmAAT* transcript abundance to the extent we achieved is by itself insufficient to further enhance SCN resistance.

**Figure 2:**
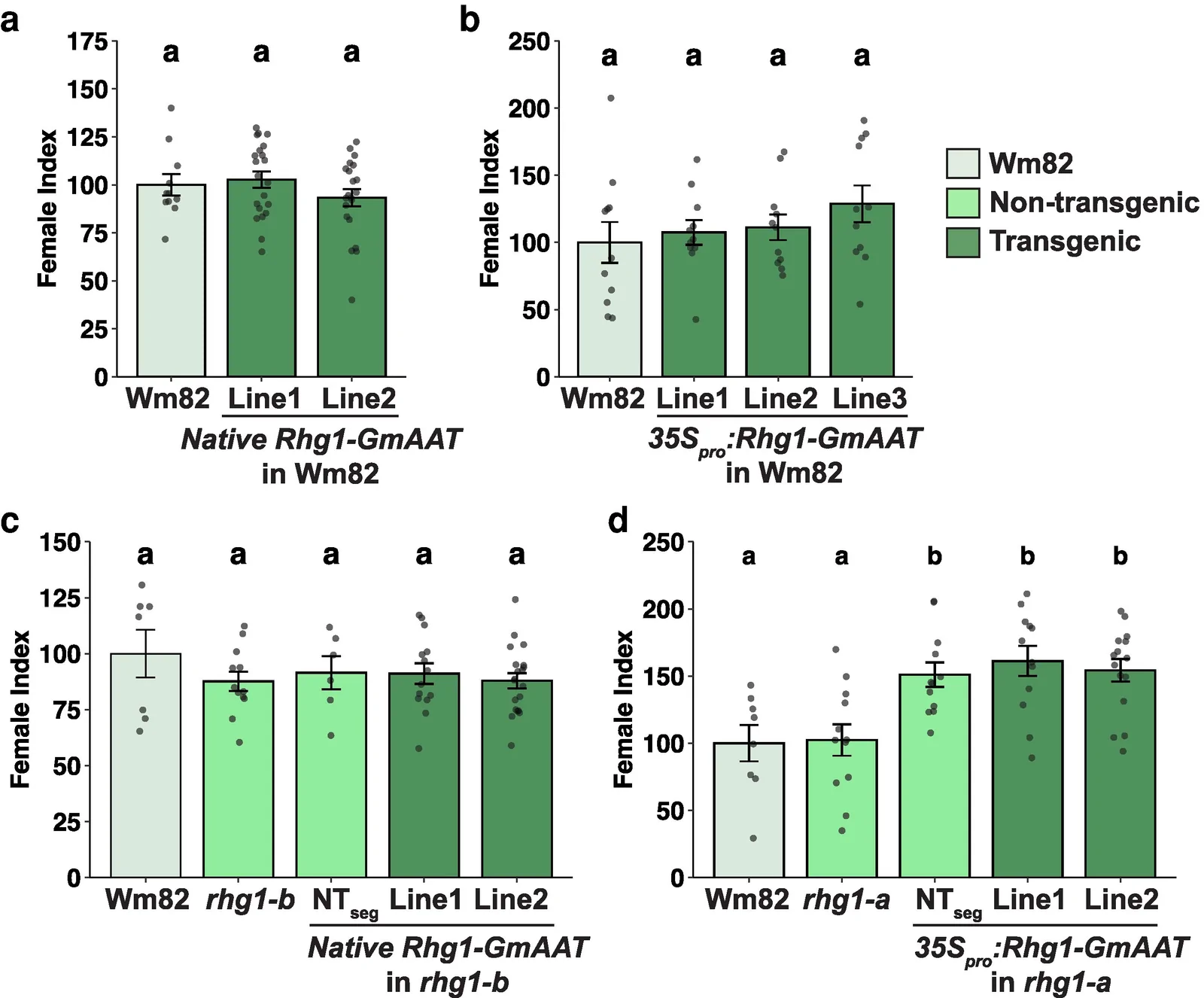
Overexpression of *Rhg1-GmAAT* did not enhance SCN resistance. Soybean lines transgenically expressing *Rhg1-GmAAT* were tested against compatible SCN populations. *Rhg1-GmAAT* transcript levels for each line are shown in Figure S4. Unless otherwise noted, transgenic lines exhibited significantly elevated *Rhg1-GmAAT* transcript levels relative to non-transgenic controls. a) SCN HG 0 resistance tests (*n* ≥ 11 for each treatment) on Wm82 transformed with a native *Rhg1-GmAAT* construct (native promoter, UTRs, exons, introns, and terminator). Line 2 showed an 11-fold but non-significant increase in transcript level (*P* = 0.1). b) SCN HG 0 resistance tests (*n* ≥ 11 for each treatment) on Wm82 transformed with an overexpression construct (*35S promoter:Rhg1-GmAAT CDS*). c) SCN HG 2.5.7 resistance tests (*n* ≥ 6 for each treatment) on IL3025N (*rhg1-b*) soybean transformed with the native construct. Controls include non-transgenic *rhg1-b* and segregated non-transgenic progeny from transformed plants (NT_seg_). Line 1 showed no increase in *Rhg1-GmAAT* expression, and Line 2 exhibited a 9-fold but marginally significant increase (*P* = 0.07). d) SCN HG 1.3.6.7 resistance tests (*n* ≥ 8 for each treatment) on IL3849N (*rhg1-a*) soybean transformed with the *35S* overexpression construct, with non-transgenic and NT_seg_ controls. For all panels, the female index was calculated relative to Wm82. Data were analyzed using ANOVA with post hoc Tukey's HSD. Bars sharing the same letter within a graph are not significantly different. Data are presented as mean ± SE. Each dot represents data from an individual plant.

### Overexpression of *Rhg1-GmAAT* or loss of *AtAVT6C* in Arabidopsis did not alter BCN resistance

A previous study showed that co-overexpression of the 3 soybean *Rhg1* genes in Arabidopsis enhanced resistance against BCN (Butler et al. 2019). To determine whether overexpression of *Rhg1-GmAAT* alone could confer BCN resistance in Arabidopsis, we transformed Arabidopsis Col-0 plants with the 2 *Rhg1-GmAAT* expression constructs described above. Stable homozygous transgenic lines were selected, and *Rhg1-GmAAT* transcript levels were tested (Figure S5a). These transgenic Arabidopsis lines did not show differences in BCN susceptibility compared with empty vector control lines (Figure S5b).

*AtAVT6C* is the ortholog of *Rhg1-GmAAT* in *A. thaliana*. A Col-0 *AtAVT6C* Arabidopsis knockout line was obtained and validated by PCR-based genotyping (Figure S6a). Subsequent BCN infection assays showed no significant differences in susceptibility between the knockout mutant and wild-type Col-0 accession (Figure S6b).

### AAT_Rhg1_ protein colocalizes with tonoplast marker VAMP711 in living soybean root cells

AAT_Rhg1_ protein was predicted to have a vacuolar membrane (tonoplast) or cell membrane localization by DeepLoc-2.1 (Ødum et al. 2024), with its N-terminus facing the cytosol according to DeepTMHMM prediction (Hallgren et al. 2022). To experimentally test the predicted tonoplast location of AAT_Rhg1_, mWasabi-tagged AAT_Rhg1_ was co-expressed in soybean root cells with a frequently used tonoplast marker, mCherry-tagged VAMP711 (Geldner et al. 2009). The tonoplast localization of mCherry-VAMP711 was verified in soybean roots (Figure S7). In root regions with nearly fully elongated cells, the tonoplast was clearly distinguished from the plasma membrane by the surrounding soluble cytosolic mWasabi (Fig. 3a). AAT_Rhg1_ was tagged with mWasabi either on the loop between the transmembrane helices TM6 and TM7 (AAT_Rhg1_ loop mWasabi) or on its N-terminus (AAT_Rhg1_ N-mWasabi). In soybean roots, AAT_Rhg1_ loop mWasabi primarily colocalized with mCherry-VAMP711 at the tonoplast, indicating vacuole membrane localization (Fig. 3a). AAT_Rhg1_ N-mWasabi also showed partial colocalization with mCherry-VAMP711 but substantially colocalized with TdTomato fused to an endoplasmic reticulum retention signal (TdTomato-ER), suggesting that N-terminal tagging of AAT_Rhg1_ can affect its subcellular trafficking (Fig. 3b). Non-transgenic soybean roots and roots expressing only mCherry or mWasabi were imaged as controls for autofluorescence and channel bleed-through (Figure S7).

**Figure 3:**
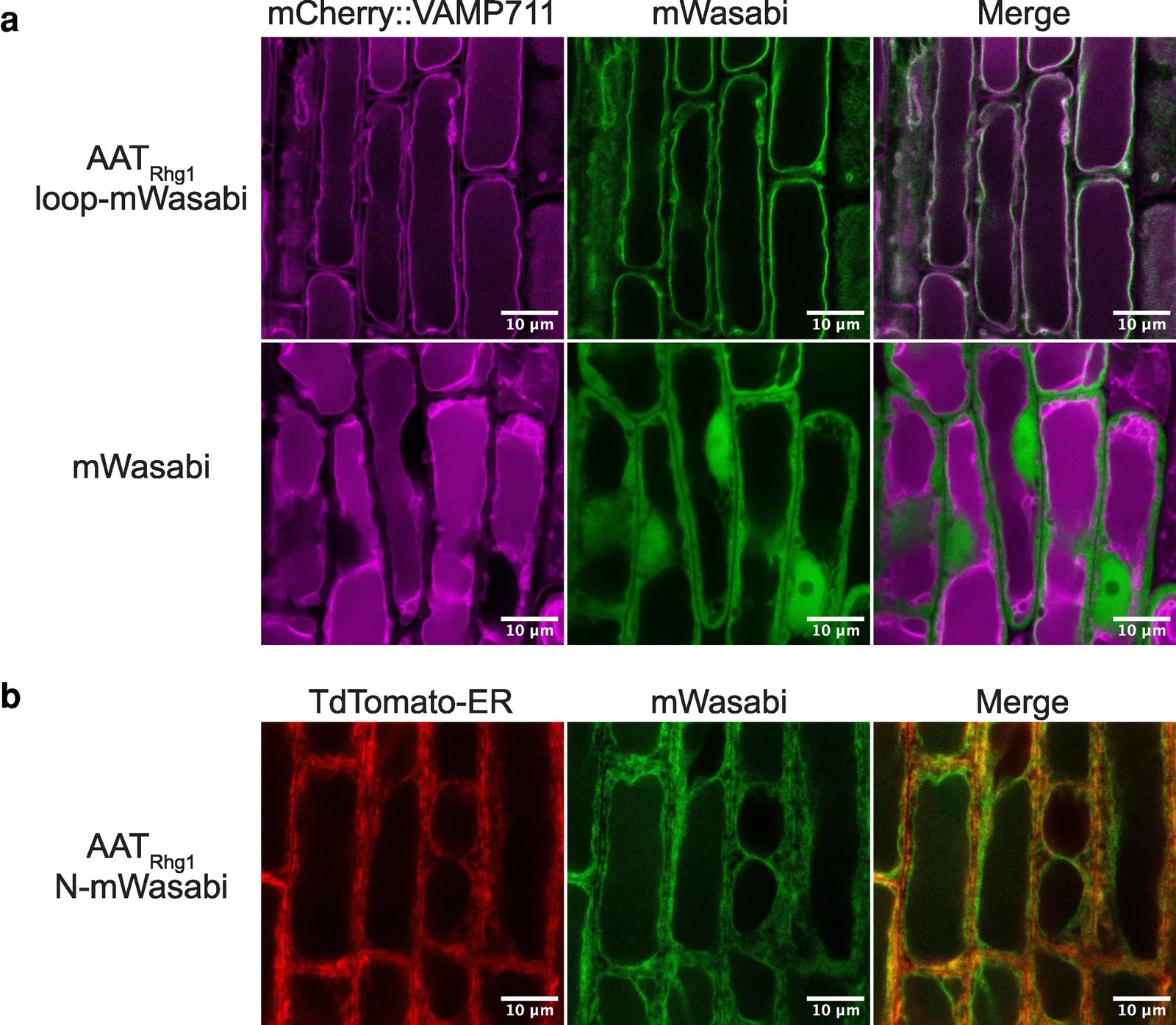
mWasabi-tagged AAT_Rhg1_ protein localizes to the tonoplast in soybean root cells. a) Representative confocal microscopy images of transgenic soybean roots co-expressing the tonoplast marker mCherry::VAMP711 and either AAT_Rhg1_ with mWasabi inserted in the loop between transmembrane helices TM6 and TM7 (loop mWasabi) or a soluble mWasabi control. Images shown are from the root zone containing nearly fully elongated root cells. White pixels in the merged images indicate colocalization. Scale bars = 10 µm. b) Representative confocal microscopy images of transgenic soybean root co-expressing TdTomato with an endoplasmic reticulum retention signal (TdTomato-ER) and N-terminally mWasabi-tagged AAT_Rhg1_ (AAT_Rhg1_ N-mWasabi). Yellow pixels in the merged images indicate colocalization. Scale bars = 10 µm.

### Silencing *Rhg1-GmAAT* affects amino acid homeostasis

Overexpression of *Rhg1-GmAAT* has been shown to affect amino acid homeostasis in soybean roots (Guo et al. 2019; Du et al. 2025). To examine the impact of silencing *Rhg1-GmAAT* on amino acid levels, the *rhg1-b* EV or iAAT plants described above were mock-inoculated or inoculated with HG 0 SCN, and 18 amino acids were quantified from infected root sections at 3 dpi using HILIC-Z LC-MS (Fig. 4; Figure S8). In the absence of SCN, silencing *Rhg1-GmAAT* resulted in significantly higher levels of leucine, isoleucine, and tyrosine (Fig. 4). In the presence of SCN at 3 dpi, isoleucine and tyrosine levels were significantly higher in iAAT roots than in EV roots (Fig. 4; *P* = 0.066 for leucine). Phenylalanine and methionine showed a similar but non-significant trend (Figure S8). Other quantified amino acids showed no significant differences between EV and iAAT plants (Figure S8). Aspartic acid and glutamic acid levels increased in response to SCN infection in both genotypes, with no observed impact from silencing *Rhg1-GmAAT* (Figure S8; similar trend for Asn and Pro not statistically significant). These findings further indicate that AAT_Rhg1_ protein abundance affects certain, but not all, amino acid levels in soybean roots.

**Figure 4:**
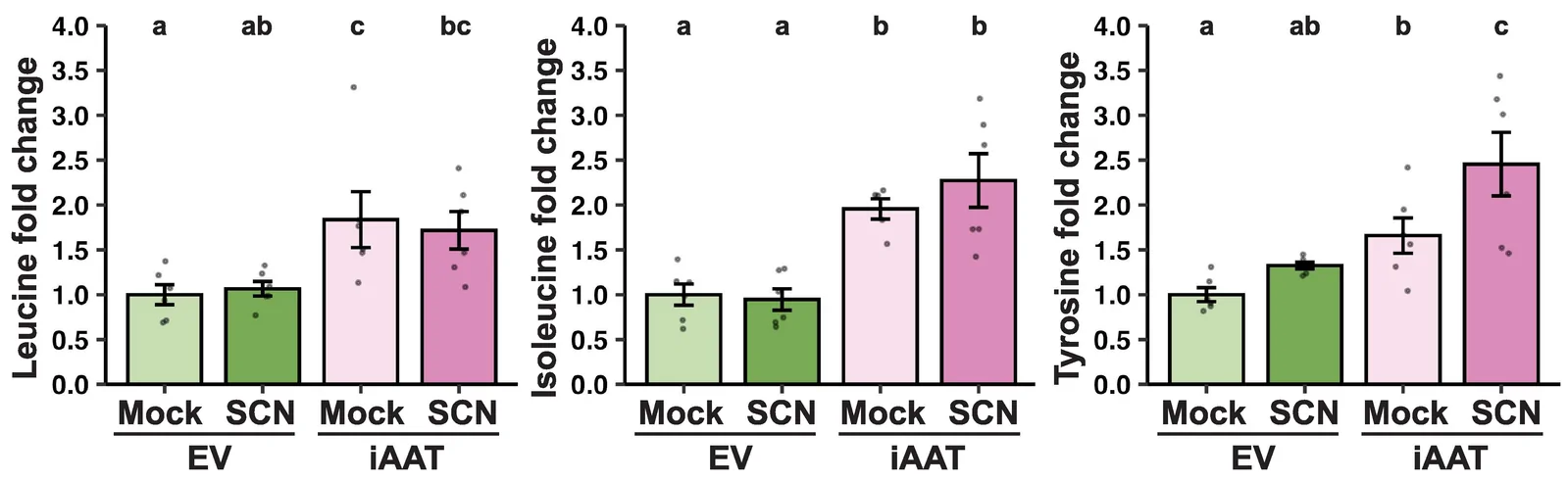
Silencing *Rhg1-GmAAT* in an *rhg1-b* background alters specific amino acid levels. Quantification of amino acid levels in roots of IL3025N (*rhg1-b*) EV or *Rhg1-GmAAT*-silenced (iAAT) transgenic soybean lines, either mock-treated or inoculated with SCN (HG 0). Samples were collected 3 d post-inoculation (dpi). Amino acid levels were normalized as fold change relative to EV-Mock samples within the same experiment. Data from 2 independent experiments were combined for analysis. Data for each amino acid were analyzed using a linear model including group and replicate as fixed effects. Bars sharing the same letter within a graph are not significantly different. Data are presented as mean ± SE (*n* = 6).

### Transcriptomic analysis reveals altered responses to SCN when *Rhg1-GmAAT* is silenced

To better understand the role of *Rhg1-GmAAT* in SCN resistance, transcriptomic analyses were performed on the *rhg1-b* EV and iAAT lines described above. Plants were either inoculated with an HG 0 SCN population or mock-inoculated. Root tissues were collected at 3 d post-inoculation (dpi) from ∼0.5 cm root sections exhibiting visible SCN infection site lesions in inoculated plants, or from corresponding root regions in mock-inoculated controls. Three dpi was chosen as a time when syncytia are established and expanding, 2 to 3 d prior to the visible syncytial collapse caused by the *rhg1-b* resistance trait (Kim et al. 2010, 2012; Mitchum 2016). Differentially expressed genes (DEGs) were identified using an adjusted *P*-value threshold of *P*_adj_ ≤ 0.05, with *P*-values adjusted using the Benjamini–Hochberg method to control the false discovery rate. Because SCN directly impacts a minority of the cells within infected root samples, no minimum transcript abundance fold-change cutoff was imposed to reduce false-negative elimination of valid DEGs whose transcript changes may be diluted by surrounding less-affected cells.

DEGs were first identified between iAAT-Mock and EV-Mock samples to investigate transcriptional changes caused by silencing *Rhg1-GmAAT* in the absence of SCN. Silencing *Rhg1-GmAAT* resulted in only 104 significantly upregulated and 78 significantly downregulated genes, suggesting minimal pleiotropic effects from reduced *Rhg1-GmAAT* expression (Figure S9a, Table S2). None of the protein-coding DEGs corresponded to known core enzymes directly involved in the biosynthesis or catabolism of leucine, isoleucine, or tyrosine. KEGG analysis revealed limited pathway enrichment among these DEGs, with only 1 group enriched to a statistically significant extent: a number of mitogen-activated protein kinase (MAPK) signaling pathway genes were significantly upregulated in iAAT-Mock relative to EV-Mock roots, all of which were associated with ethylene signaling (Figure S9b, Table S3 tab for Figure S9b).

In contrast to the mock-only comparison above, more than 5,000 DEGs were observed upon SCN infection within each genotype (SCN vs. Mock comparisons; Figure S10a and b, Table S2). KEGG enrichment analyses indicated broad transcriptional responses to SCN infection (Figure S10c to f, Table S3). For infected root areas of SCN-resistant *rhg1-b* EV plants, observed transcriptional SCN responses (Figure S10) carried multiple similarities to previously reported SCN-responsive gene expression profiles (Kandoth et al. 2011; Song et al. 2019; Wei et al. 2022; Zhang et al. 2022; Torabi et al. 2023; Sultana et al. 2024). Several KEGG pathways related to defense signaling, metabolism, and protein processing were significantly enriched among SCN-upregulated genes in *rhg1-b* EV roots but not in iAAT roots, including MAPK signaling, protein processing in ER, cyanoamino acid metabolism, and starch and sucrose metabolism (asterisks in Figure S10c).

To more stringently examine SCN-responsive transcriptional differences between *rhg1-b* EV and iAAT plants, we refined the above SCN-responsive DEG lists by separating the 2,160 upregulated and 1,007 downregulated genes shared by both plant genotypes (Fig. 5a to d;  Table S2). Those shared DEGs were enriched in KEGG pathways related to defense and metabolism, as well as circadian rhythm, DNA replication, and protein processing (Fig. 5c and d;  Table S3), suggesting that *Rhg1-GmAAT* is less central in modulating those SCN responses. Excluding the shared DEGs allowed identification of SCN-responsive genes whose expression is robustly affected by *Rhg1-GmAAT* silencing, which are listed in Table S2 on tabs whose names start with the word “Unique”. Their significantly overrepresented KEGG groups are presented in Fig. 5e to h. It remained of particular interest to identify genes upregulated in response to SCN in *rhg1-b* EV roots but not in iAAT roots. This set of genes was again enriched for MAPK signaling pathway, starch and sucrose metabolism, and a pair of overlapping KEGG gene sets related to secondary metabolite biosynthesis and cyanoamino acid metabolism (Fig. 5e; individual genes listed in Table S3 tab for Fig. 5e). Intriguingly, no KEGG groups were significantly overrepresented among EV-specific downregulated genes (Fig. 5f). In the more SCN-susceptible iAAT roots, genes uniquely upregulated during SCN infection were associated with motor proteins, proteasome, and DNA replication (Fig. 5g), whereas genes uniquely downregulated in iAAT roots were significantly enriched in metabolic and stress-response pathways: MAPK signaling, branched-chain amino acid (Val/Leu/Ile) degradation, carotenoid biosynthesis, and autophagy (Fig. 5h).

**Figure 5:**
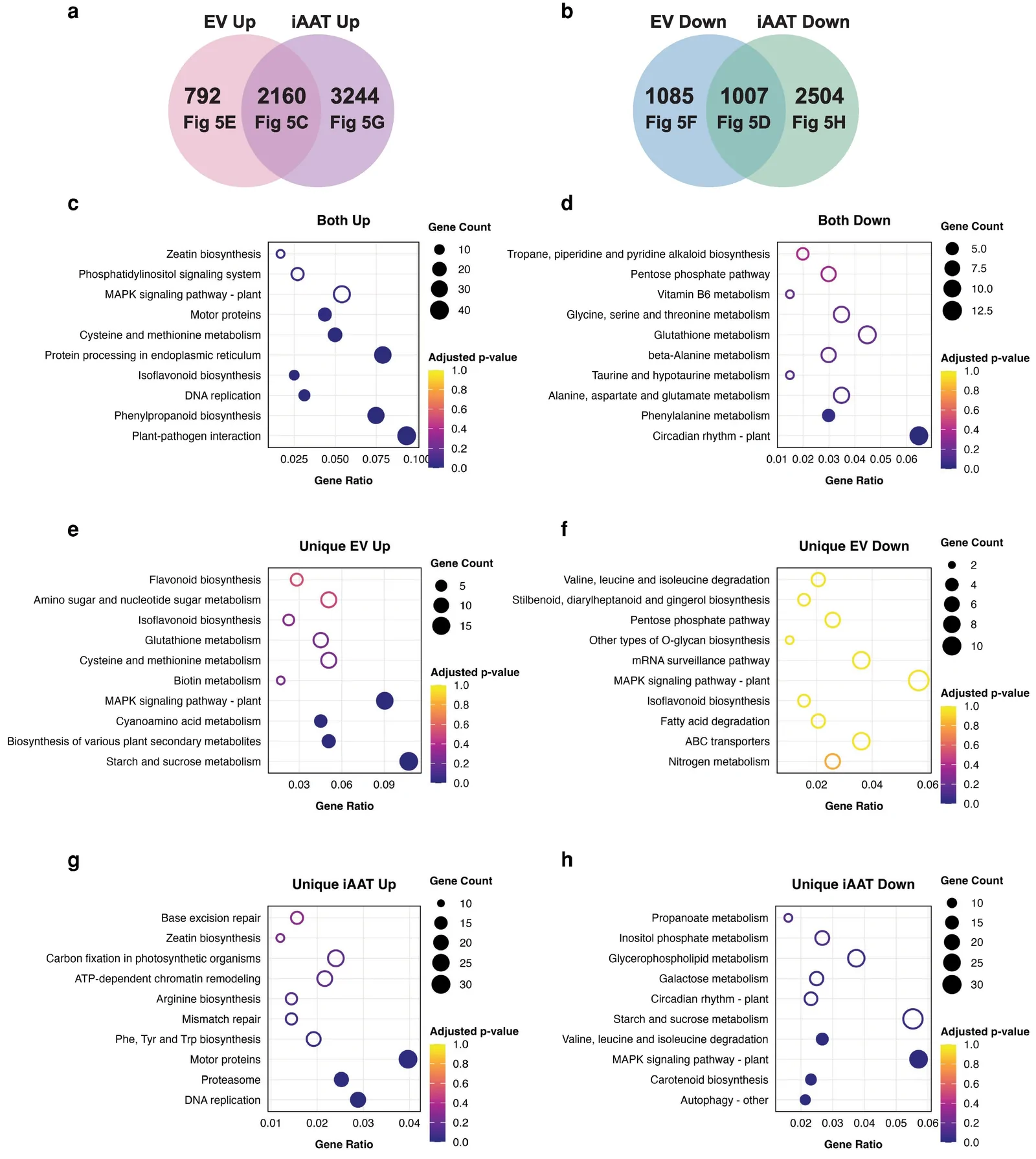
Shared and genotype-specific transcriptional responses to SCN infection in EV and iAAT soybean lines. a, b) Venn diagrams showing overlaps of SCN-responsive DEGs between EV and iAAT lines. The numbers of upregulated genes are shown in (a), and downregulated genes in (b). c to h) KEGG pathway enrichment dot plots for DEGs shared between or unique to genotypes. DEGs shared by both genotypes are shown in c) for upregulated genes and d) for downregulated genes. EV-specific DEGs are shown in e) for upregulated and f) for downregulated genes. iAAT-specific DEGs are shown in g) for upregulated and h) for downregulated genes. Dot size represents the number of DEGs associated with each pathway. Filled circles illustrate significantly enriched pathways (adjusted *P*-value < 0.05); empty circles indicate non-significant enrichment.

Given that MAPK signaling pathway genes (KEGG ID gmx04016) were significantly overrepresented as being upregulated in response to SCN infection uniquely in SCN-resistant *rhg1-b* EV roots, and downregulated uniquely in iAAT roots, we further examined this finding and discovered a high presence of ethylene-associated genes. Nine of the 16 genes on the MAPK signaling pathway KEGG list for the “Unique EV Up” set of DEGs are annotated for ethylene synthesis, perception or responses (Fig. 6; Table S3 tab for Fig. 5e). An additional 11 ethylene-associated genes were also in that “Unique EV Up” DEG group but are not annotated as part of the KEGG MAPK signaling pathway (Fig. 6). Intriguingly, in the “Unique iAAT Down” set, 16 of 32 DEGs in the KEGG MAPK signaling pathway (Fig. 5h) were also ethylene-associated genes, as were an additional 24 genes in the “Unique iAAT Down” DEG set (Figure S11; Table S3 tab for Fig. 5h). AAT_Rhg1_ apparently makes a significant contribution to *rhg1-b* ethylene-mediated responses to SCN infection.

**Figure 6:**
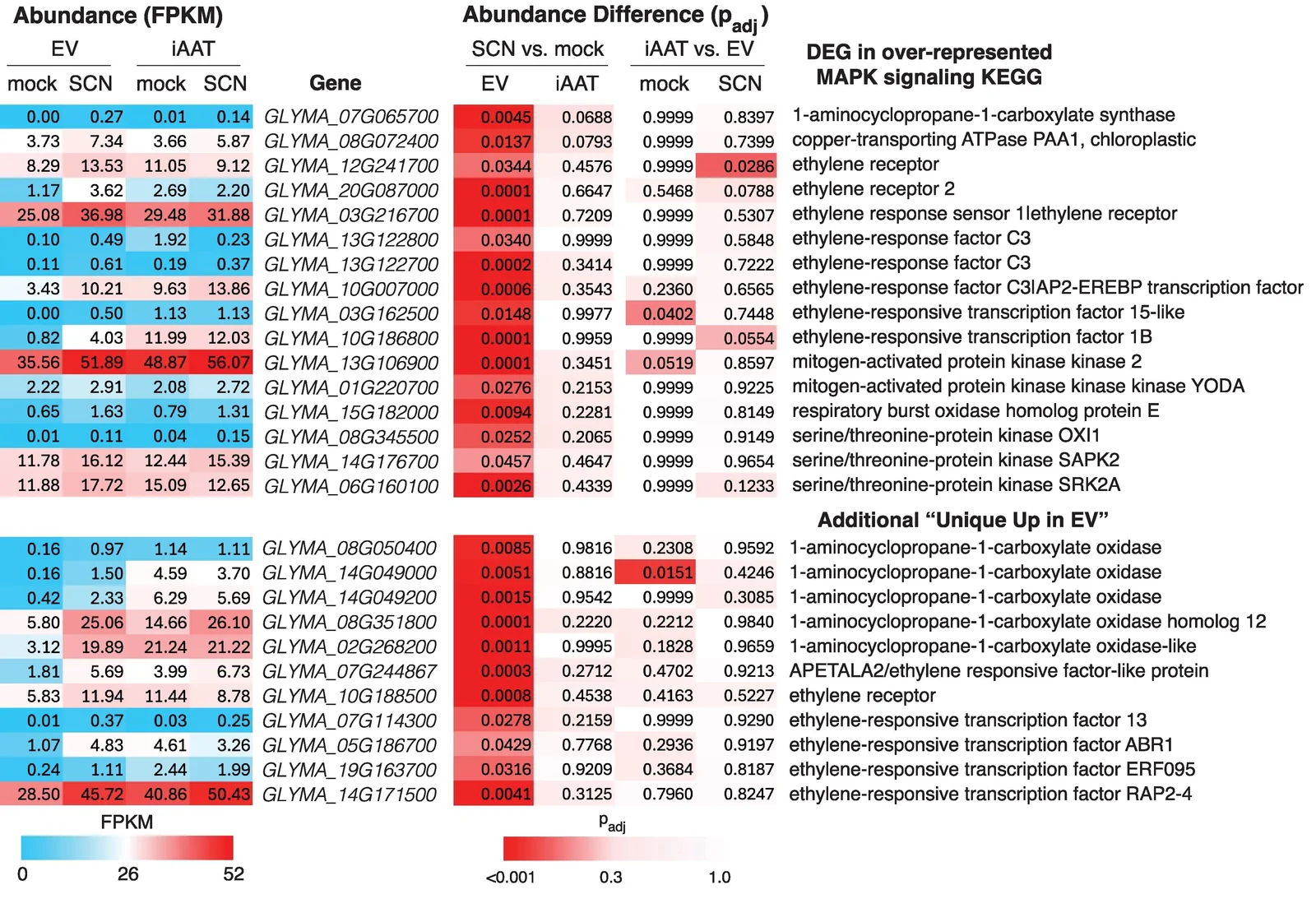
Silencing of *Rhg1-GmAAT* significantly alters abundance of transcripts associated with MAPK signaling and ethylene responses. Top: Transcripts significantly elevated at SCN infection sites in SCN-resistant *rhg1-b* (EV) roots but not in *Rhg1-GmAAT*-silenced roots (iAAT), for the overrepresented KEGG group “MAPK signaling pathway—plant”. Bottom: Additional genes uniquely up in EV but not iAAT roots in response to SCN that carry ethylene-associated annotation but are not placed by KEGG into the above group.

While the above KEGG analyses identify broad pathway trends, it should be noted that multiple individual genes with potentially high biological relevance were not highlighted. The complete lists of DEGs are provided in Table S2.

### Untargeted metabolomic profiling indicates broad metabolic changes in *Rhg1-GmAAT*-silenced soybean roots

To investigate metabolomic changes associated with *Rhg1-GmAAT* silencing, we performed untargeted metabolomic analysis on root samples from EV and iAAT plants, either mock-inoculated or inoculated with SCN at 3 dpi. Principal component analysis (PCA) showed that silencing of *Rhg1-GmAAT* substantially affected the levels of detectable metabolites, with a stronger overall effect on metabolomic profiles than SCN infection itself (Fig. 7a).

**Figure 7:**
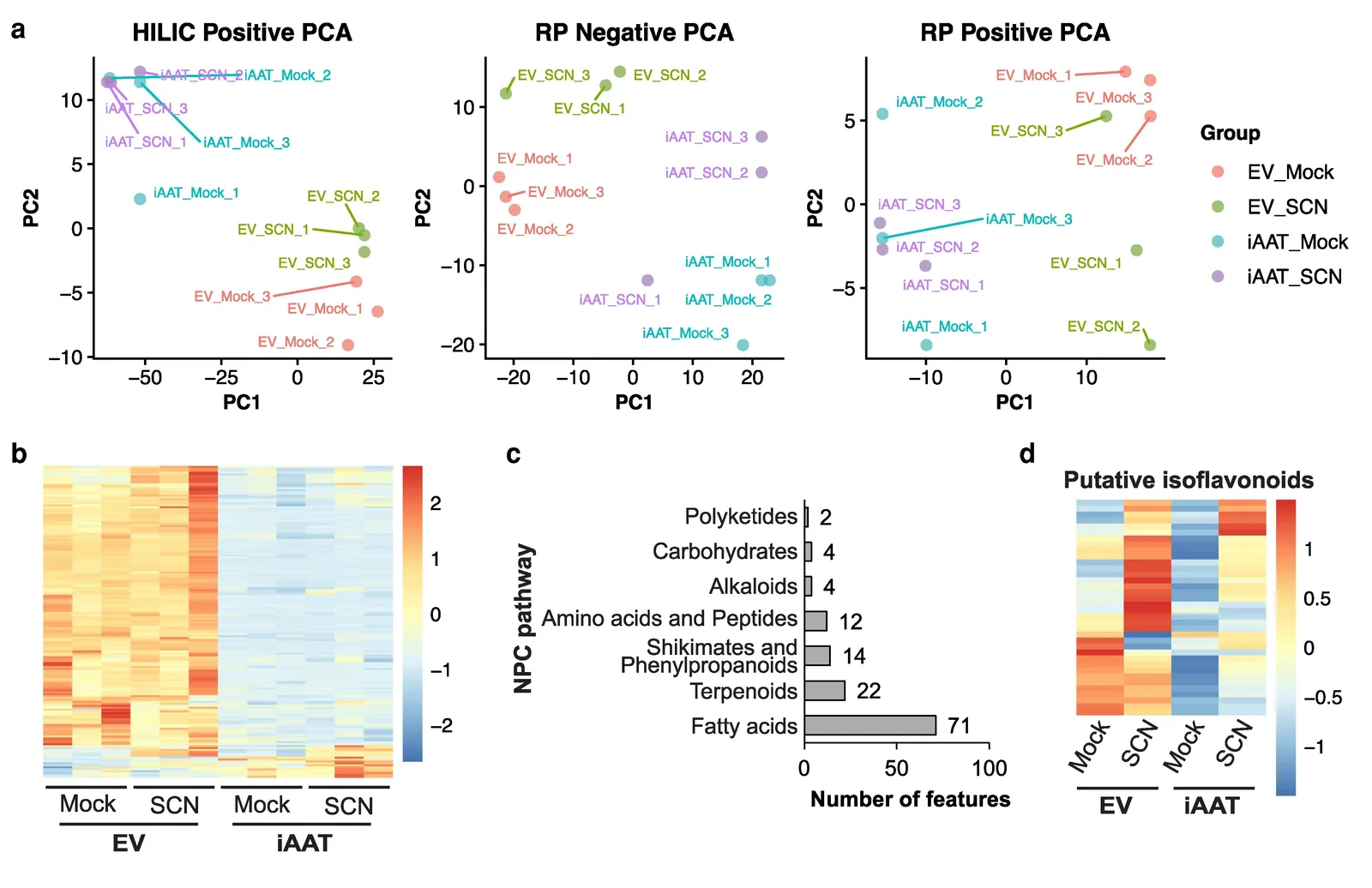
Silencing *Rhg1-GmAAT* in an *rhg1-b* background alters metabolomic profiles. a) PCA of untargeted metabolomic profiles obtained using HILIC (positive mode) and RP chromatography (positive and negative modes). Metabolomic mass features were quantified from root infection zones of *rhg1-b* EV or iAAT transgenic soybean lines, either mock-inoculated or inoculated with SCN (HG 0) at 3 dpi. b) Heatmap showing mass features that were significantly altered (fold-change >2 or <0.5; *t*-test *P* < 0.05) in at least 1 pairwise comparison between EV-mock vs. iAAT-mock or EV-SCN vs. iAAT-SCN detected with RP-negative mode. c) NPC pathway classification of the differentially abundant mass features in (b) with available NPC annotations. Bars indicate the number of mass features assigned to each NPC biosynthetic pathway. d) Heatmap showing the relative abundances of predicted isoflavonoids detected in RP-negative mode.

To further characterize these metabolic shifts, we analyzed LC-MS data using MZmine (Schmid et al. 2023), followed by molecular structure prediction in SIRIUS (Dührkop et al. 2019). Because RP-negative-mode ionization yielded overall higher compound prediction confidence, subsequent analyses focused on this dataset. In total, 612 mass features were detected, of which 412 were assigned predicted molecular structures (Table S4). Among all detected features, 162 (26%) showed significant abundance changes (fold-change >2 or < 0.5; *t*-test *P* < 0.05) in at least 1 pairwise comparison between EV-mock vs. iAAT-mock or EV-SCN vs. iAAT-SCN (Fig. 7b). To gain insight into the metabolic categories affected by *Rhg1-GmAAT* silencing, annotated compounds were classified using the natural product classification (NPC) system (Kim et al. 2021). Among the significantly altered features, fatty acids represented the largest category, followed by terpenoids, shikimates and phenylpropanoids, and amino acid-derived metabolites (Fig. 7c;  Figure S12).

Isoflavonoids have been implicated previously in soybean resistance to SCN (Jiang et al. 2025), and genistein and daidzein are recognized as soybean phytoalexins due to their antimicrobial activities (Dakora and Phillips 1996). Analysis of predicted isoflavonoid-related features from the RP-negative-mode dataset showed an overall trend toward reduced abundance in iAAT roots compared with EV roots, in both mock-treated and SCN-infected tissues, with particularly low isoflavonoid levels in mock-treated iAAT roots (Fig. 7d; Table S4).

### KEGG-based association of transcriptomic and metabolomic responses

To examine associations between SCN-responsive transcriptomic changes and metabolomic changes, we performed a KEGG-based transcriptome-metabolome convergence analysis. KEGG enrichments of SCN-responsive genes and metabolites in EV and iAAT were compared to identify pathways supported by both omics datasets. Isoflavonoid biosynthesis was identified as the only pathway showing transcriptome-metabolome convergence (Table S4; Figure S13). We therefore mapped KEGG-associated genes and KEGG-mapped metabolite features onto a simplified KEGG gmx00943 pathway. This visualization showed broad SCN-responsive changes in both EV and iAAT roots, while also highlighting genotype-dependent differences in pathway gene induction and branch-specific metabolite responses (Figure S14).

## Discussion

Our results show that *Rhg1-GmAAT* can contribute to resistance against not only HG 0, but also a more virulent HG 2.5.7 SCN population that partially overcomes the resistance conferred by *rhg1-b*. The HG 2.5.7 MN population may have evolved mechanisms to escape the quantitative resistance conferred via the other 2 *Rhg1*-encoded proteins but not AAT_Rhg1_. Alternatively, it may partially overcome the resistance conferred by AAT_Rhg1_, but in either case, AAT_Rhg1_ still provides quantitative resistance. Because of genetic heterogeneity within SCN populations, it is also possible that only some individuals are capable of overcoming AAT_Rhg1_. The observed differences between HG 2.5.7 MN and HG 2.5.7 MO populations further suggest that SCN populations classified under the same HG type may have evolved through distinct paths, leading to variations in their ability to reproduce on a resistant soybean line.

Root overexpression of just the *Rhg1-GmAAT* gene, via the CaMV *35S* promoter or additional native-promoter copies, was insufficient to enhance resistance as measured in the present study, regardless of SCN resistance background. Because the CaMV *35S* promoter does not reproduce the native infection-induced expression pattern of *Rhg1-GmAAT*, the negative results from the promoter-driven lines could not exclude the possibility that properly timed and localized *Rhg1-GmAAT* expression may enhance SCN resistance. Some reports suggest that some *35S* promoter constructs are not expressed well in syncytia at multi-week time points (Urwin et al. 1997; Bertioli et al. 1999; Ali and Abbas 2016). We did not specifically pursue a syncytium-focused promoter strategy because our prior work showed that AAT_Rhg1_ protein does not show pronounced accumulation in the syncytium, and instead accumulates primarily along the SCN penetration path (Han et al. 2023). A previous study using detached roots also did not detect increased resistance from *35S* promoter-driven *Rhg1-GmAAT* overexpression (Liu et al. 2017). Use of other promoters to drive *Rhg1-GmAAT* expression could prove successful where our efforts were not.

In contrast, elevated expression of α-SNAP_Rhg1_ has been shown to quantitatively enhance resistance against SCN, although the efficacy depended on α-SNAP_Rhg1_ isoform, *Rhg1* haplotype, soybean genetic background, and SCN population (Liu et al. 2017; Haarith et al. 2025). Co-overexpression of the 3 *Rhg1* genes in soybean has been shown to promote resistance against SCN in transgenic Wm82 roots (Cook et al. 2012). Beyond soybean, transformation of Arabidopsis and potato plants with constructs that direct overexpression of the 3 *Rhg1* genes also elevated resistance, in that case against BCN and potato cyst nematodes (*Globodera rostochiensis* and *Globodera pallida*) (Butler et al. 2019). To date, AAT_Rhg1_ itself has been shown to be necessary but not sufficient for full resistance. These findings support the hypothesis that the 3 *Rhg1* genes function synergistically, with all 3 required to trigger full SCN resistance. It is possible that AAT_Rhg1_ contributes to a broader physiological or metabolic context that facilitates resistance responses, such as maintaining amino acid or phytohormone homeostasis, priming phytoalexin biosynthesis, or activation of defense signaling cascades. These pathways may not be fully engaged without coordinated tissue- and infection-appropriate expression of AAT_Rhg1_ along with the other 2 *Rhg1* proteins α-SNAP_Rhg1_ and WI12_Rhg1_, which could explain the lack of enhanced resistance in *Rhg1-GmAAT* overexpression lines.

We investigated the effects of the AAT_Rhg1_ conserved-site mutations Y268L and D122A on SCN resistance. We recently reported the synthetic phenotype that AAT_Rhg1_ overexpression enhances *RUBY*-mediated betalain production in soybean roots, while AAT_Rhg1_ D122A fails to do so and AAT_Rhg1_ Y268L causes even stronger pigmentation (Du et al. 2025). In the present work, synthesized RNAi-insensitive *SynAAT_Rhg1_WT* successfully restored SCN resistance in an iAAT soybean line. Consistent with the betalain phenotype, *SynAAT_Rhg1_D122A* failed to complement SCN resistance. The Y268 residue is predicted to reside within the internal channel of AAT_Rhg1_ near its putative substrate-binding site, and similar mutations in other amino acid transporters have been shown to alter transport activity (Errasti-Murugarren et al. 2019). *SynAAT_Rhg1_Y268L* also restores SCN resistance, but unlike its stronger effect on betalain overproduction, *SynAAT_Rhg1_Y268L* did not cause greater SCN resistance than wild-type. These results suggest that although AAT_Rhg1_Y268L may enhance cytosolic tyrosine availability for betalain synthesis, this does not translate into an overt increase in SCN resistance. AAT_Rhg1_-mediated SCN resistance may be independent of its putative amino acid transport function and instead involve other activities of the protein such as protein-protein interactions. Alternatively, Y268L might represent a more active form of AAT_Rhg1,_ but substrate transport may not be limiting for SCN resistance, analogous to overexpressing wild-type AAT_Rhg1_ in an *rhg1-b* background, which also did not confer further resistance.

This study revealed that AAT_Rhg1_ protein localizes to the tonoplast in intact soybean root cells, where it likely functions as a transporter and regulates amino acid homeostasis. The addition of an N-terminal GFP tag disrupted AAT_Rhg1_ tonoplast localization and resulted in ER retention, suggesting that the N-terminus may contribute to proper subcellular targeting of AAT_Rhg1_, as the first transmembrane domain in many membrane proteins often functions as a signal anchor (Wu and Hegde 2023). In damaged cells along the SCN penetration path, AAT_Rhg1_ has also been observed in macrovesicle-like membrane structures (Han et al. 2023). These structures may originate from the tonoplast of cells disintegrated by SCN penetration, and although the entire cell is no longer intact, the macrovesicles could still retain biochemical or signaling functions. At the cyst nematode feeding syncytium, the large tonoplast-bounded central vacuole degenerates and the cytoplasm fills with multiple much smaller vacuole structures (Endo 1965; Mitchum 2016). This may partly explain the absence of pronounced AAT_Rhg1_ protein abundance elevation in the syncytium (Han et al. 2023), in contrast to another *Rhg1*-encoded protein, α-SNAP_Rhg1_, which specifically accumulates in the syncytium (Bayless et al. 2016). The tonoplast location of AAT_Rhg1_ on a cellular structure that undergoes such dramatic remodeling during SCN parasitism may anticipate intriguing, currently unknown contributions of AAT_Rhg1_ to resistance.

Although the amino acid transporter activity of AAT_Rhg1_ has not been mechanistically demonstrated, differences in *Rhg1-GmAAT* expression influence amino acid homeostasis in soybean root cells. Yeast (*Saccharomyces cerevisiae*) *AVT6* encodes a yeast vacuolar amino acid transporter that shares structural similarities with AAT_Rhg1_ (Russnak et al. 2001; Chahomchuen et al. 2009), and interestingly, AVT6 was associated with higher tyrosine production (Wu et al. 2020). Silencing *Rhg1-GmAAT* altered the abundance of leucine, isoleucine, and tyrosine in whole-cell extracts, showing an overall trend opposite to that observed in *Rhg1-GmAAT* overexpression roots (Guo et al. 2019; Du et al. 2025). These observations suggest that precise control of *Rhg1-GmAAT* expression may be important for maintaining homeostasis of certain amino acids under both basal and stress conditions. Direct identification of the amino acid substrates of AAT_Rhg1_ and mechanistic demonstration of its transport activity may require future assays that preserve the native membrane context of this tonoplast-localized protein.

In *Rhg1-GmAAT*-silenced roots, genes encoding canonical biosynthetic or catabolic enzymes for leucine, isoleucine, or tyrosine were not significantly differentially expressed compared with EV roots under mock-treated conditions, suggesting that 3 dpi transcript abundance changes alone do not directly explain the differences in amino acid levels. This discrepancy could reflect transient transcriptional changes not captured at our sampling time point, post-transcriptional regulation, or effects on subcellular amino acid distributions and sequestration. We quantified amino acids from whole-cell/whole-tissue extracts, and it remains possible that AAT_Rhg1_ also affects subcellular or spatially restricted amino acid pools that are critical for local defense activation. The findings that AAT_Rhg1_ localizes to the tonoplast and influences amino acid homeostasis raise the possibility that altered distribution of specific amino acids between the vacuole and the cytosol, and/or vacuole-derived membrane structures at the root–nematode interface, could contribute to defense against SCN. Future studies that resolve vacuolar vs. cytosolic amino acid partitioning may also help clarify how AAT_Rhg1_ influences local amino acid homeostasis during SCN infection.

Plant amino acid homeostasis and amino acid transporters participate in defense signaling during biotic stress (Sonawala et al. 2018; Yao et al. 2020). A recent study has shown that microbe-associated molecular pattern perception induces accumulation of apoplastic amino acids, suppressing bacterial virulence in Arabidopsis (Zhang et al. 2023). Arabidopsis amino acid permease gene family members have been linked to susceptibility to BCN and root-knot nematode (*Meloidogyne incognita*) (Elashry et al. 2013; Marella et al. 2013; Dutta et al. 2024). Arabidopsis *Lysine Histidine Transporter 1* mediates cellular and extracellular amino acid contents and participates in regulating defense against pathogens (Liu et al. 2010; Rogan et al. 2024). Although the present study does not identify a specific mechanism by which altered amino acid levels stimulate defense, our findings with AAT_Rhg1_ are consistent with a general model that plants may sense and incorporate inappropriate amino acid levels as part of their defense signaling network.

Our transcriptomic analyses revealed that silencing *Rhg1-GmAAT* substantially alters SCN-induced gene expression profiles, impacting transcripts for multiple metabolic and defense-related pathways, any of which may be related to SCN resistance. Relative to SCN-resistant *rhg1-b* EV roots, iAAT roots exhibited more extensive transcriptional SCN responses (a greater number of DEGs). This may be related to increased nematode parasitism in plants with less resistance. Multiple MAPK signaling DEGs and especially ethylene signaling-related DEGs were upregulated more in *rhg1-b* EV than in iAAT roots, indicating that AAT_Rhg1_ influences basal defense stress signaling, possibly priming or modulating host readiness to respond to SCN infection.

The present study found downregulation of many ethylene-associated transcripts in the more SCN-susceptible iAAT line, in addition to upregulation of other ethylene-associated transcripts in the more SCN-resistant EV (*rhg1-b*) line. In Arabidopsis–BCN interactions, nematode penetration elicits an ethylene response, and feeding site establishment was more successful in ethylene signaling-deficient mutants (Marhavý et al. 2019). Other transcriptomic studies have highlighted DEGs involved in ethylene biosynthesis and signaling at the early stages of cyst nematode infection, which may be related to SCN resistance in various soybean genetic backgrounds (Miraeiz et al. 2020; Chaiprom et al. 2024). Notably, ethylene was recently demonstrated to be a positive regulator of the expression of 2 *Rhg1* genes, *Rhg1-GmAAT* and *GmSNAP18*, and to contribute to SCN resistance in an *rhg1-a* background (He et al. 2025). Additionally, ethylene-responsive transcription factors have been shown to activate the expression of downstream defense genes in response to SCN infection (Mazarei et al. 2002, 2007; Deng et al. 2024). But contrary to the above, in Arabidopsis, ethylene has been implicated in beet cyst nematode attraction to roots and in nematode susceptibility (Wubben et al. 2001; Kammerhofer et al. 2015; Piya et al. 2019), and an ethylene-insensitive soybean line exhibited reduced infection by SCN (Bent et al. 2006). Some of the DEGs downregulated in iAAT roots, which were more susceptible to SCN, are annotated as EIN3-binding F-box proteins whose reduced abundance could increase ethylene signaling. In the present study, the differential impacts of *Rhg1-GmAAT* on numerous ethylene-associated transcripts collectively implicate AAT_Rhg1_ as a modulator of ethylene signaling during defense against SCN.

Overall, as is often true with RNA-seq study results, major challenges going forward are to identify and further investigate the most salient leads. The choice of leads may differ depending on whether the priority is to study broader aspects of plant susceptibility to SCN rather than to identify the AAT_Rhg1_ activities that most directly modulate SCN resistance.

Metabolomics analysis showed that silencing *Rhg1-GmAAT* altered the metabolite profile in soybean roots, with a more extensive overall impact than SCN infection, suggesting this putative amino acid transporter contributes to aspects of root metabolic homeostasis beyond its role in mediating SCN resistance. Among the significantly altered mass features detected by reversed-phase negative-mode LC-MS, fatty acids, terpenoids, shikimate/phenylpropanoid compounds, and amino acid derivatives were the most represented NPC pathway categories. Within these groups, predicted flavonoids and isoflavonoids, terpenoids, and jasmonic acid-related intermediates were notable due to their previously reported association with plant–nematode interactions (Dakora and Phillips 1996; Chin et al. 2018; Guo et al. 2019; Meresa et al. 2024; Jiang et al. 2025). These metabolomic changes offer potential biochemical context for the reduced SCN resistance observed in *Rhg1-GmAA*T-silenced plants, although the specific contributions of individual metabolites remain to be clarified. Our RNA-seq analysis did not identify consistent changes in the expression of major biosynthetic genes that would readily account for these metabolic differences, suggesting that the observed metabolite alterations may arise from mechanisms other than large transcriptional reprogramming, such as changes in substrate availability or pathway flux. Additional work will be needed to determine how AAT_Rhg1_ activity influences these metabolic outcomes.

The present study defines multiple previously unknown functional parameters of AAT_Rhg1,_ including AAT_Rhg1_ subcellular location and specific impacts on pre-infection and SCN-responsive transcription and metabolic responses. Substantial work is still needed to understand the mechanisms by which AAT_Rhg1_ confers resistance. However, the present study also demonstrates that *Rhg1-GmAAT* makes significant contributions to SCN resistance against distinct SCN populations. The above insights may already assist efforts to engineer more durable SCN resistance.

## Supplementary Material

kiag706_Supplementary_Data

## Data Availability

The data underlying this article are available in the article and in its online supplementary material, or will be shared on reasonable request to the corresponding author.
