# Supplementary material for: AATRhg1 is a tonoplast protein that alters amino acid, metabolic, and defense responses and nematode resistance
Source: Plant Physiol. 2026 Sep 21;202(2):kiag706. doi: 10.1093/plphys/kiag706 (PMC13651907; doi:10.1093/plphys/kiag706)
Supplement: Supplementary file 1 [file kiag706_supplementary_data.zip › Du-SupplementalMaterials.pdf]

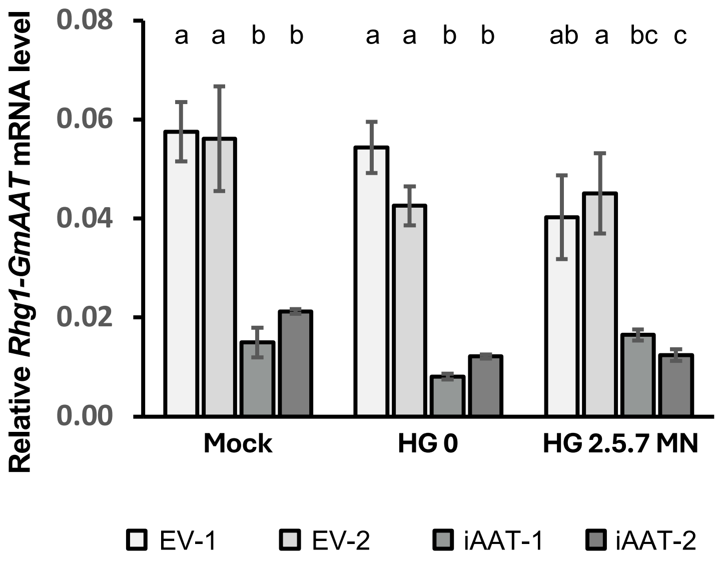

Supplemental Figure S1. *Rhg1-GmAAT* is effectively silenced in RNAi transgenic soybean lines.

Relative transcript levels of *Rhg1-GmAAT* were measured by RT-qPCR and normalized to the reference gene *GmNREG* in IL3025N (*rhg1-b*) transgenic lines transformed with either an empty vector (EV) or an *Rhg1-GmAAT* RNAi-silenced (iAAT) construct, following mock treatment or inoculation with SCN HG 0 or HG 2.5.7 from Minnesota (HG 2.5.7 MN) at 3 days post-inoculation (dpi). Data were analyzed using ANOVA with post-hoc Tukey HSD (n = 3). Bars sharing the same letter within each SCN treatment are not significantly different. Data are presented as mean ± SE.

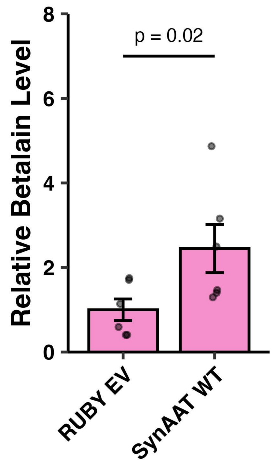

Supplemental Figure S2. Synthesized RNAi-escaping *Rhg1-GmAAT* functions in *RUBY*-mediated betalain synthesis.
Synthesized codon-substituted RNAi-escaping *Rhg1-GmAAT* wild-type (*SynAAT WT*) was co-expressed with *RUBY* in Williams 82 roots. Betalains were quantified as (A_{538} + A_{476}) per gram of fresh weight, and normalized to roots expressing *RUBY*-alone empty vector (*RUBY* EV). Data were analyzed using one-tailed *t*-test and presented as mean ± SE. Each dot represents one sample.

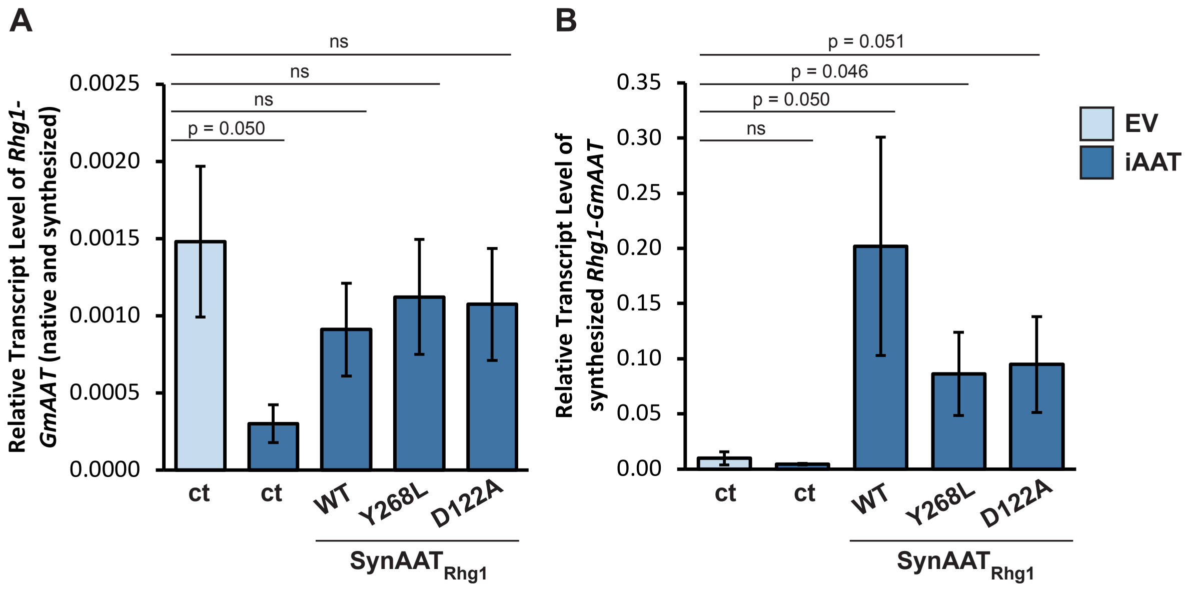

Supplemental Figure S3. Transcript levels of native and/or synthetic *Rhg1-GmAAT* in soybean roots.

Transgenic EV plants further transformed with a marker-only construct (ct) were used as a high-expression control, and transgenic iAAT plants further transformed with a marker-only construct (ct) were used as a low-expression control. Relative transcript abundance of native and/or synthesized *Rhg1-GmAAT* was measured by RT-qPCR and normalized to the reference gene *GmNREG*. Transcript levels of *SynAAT*$_{Rhg1}$ wild type (WT), *SynAAT*$_{Rhg1}$ *Y268L*, or *SynAAT*$_{Rhg1}$ *D122A* were measured using primers amplifying both native and synthetic *Rhg1-GmAAT* **(A)** or only synthetic *SynAAT*$_{Rhg1}$ **(B)**. Data were analyzed using one-tailed *t*-test and are presented as mean ± SE (n = 3 or 4).

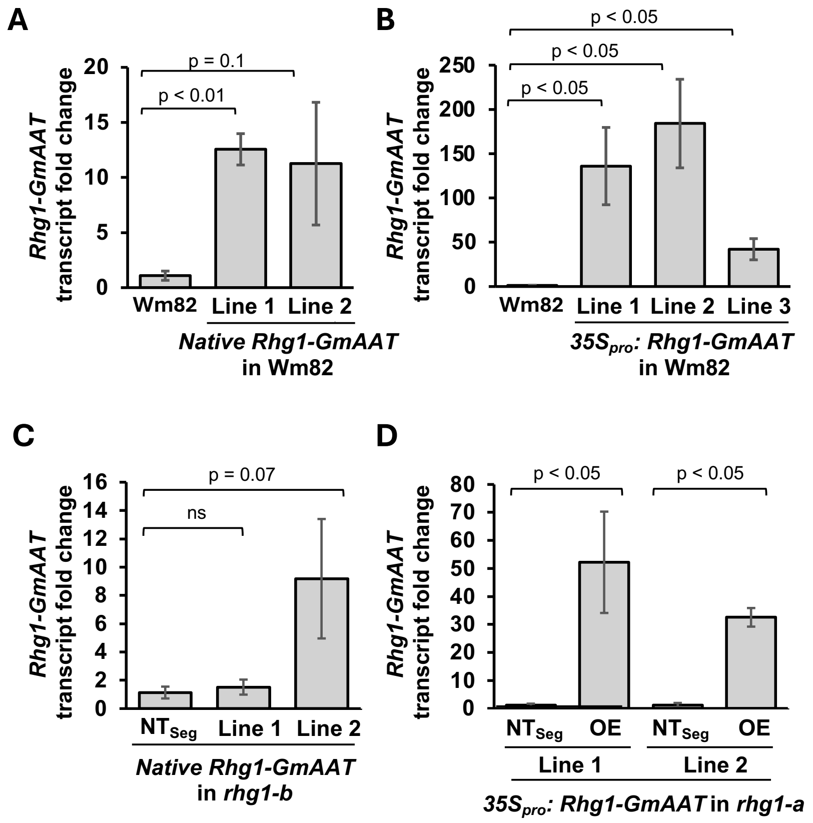

Supplemental Figure S4. Overexpression of *Rhg1-GmAAT* in transgenic soybean lines.

Transcript levels of *Rhg1-GmAAT* were measured by RT-qPCR and normalized to the reference gene *GmEF1A* in the following transgenic soybean lines:

(A) Williams 82 transformed with a native *Rhg1-GmAAT* construct (native promoter, UTRs, exons, introns and terminator)

(B) Williams 82 transformed with a *35S promoter:Rhg1-GmAAT CDS:Nos terminator* construct.

(C) IL3025N (*rhg1-b*) transformed with the native *Rhg1-GmAAT* construct. Non-transgenic progeny from transformed plants (NT_{seg}) were used as controls.

(D) IL3849N (*rhg1-a*) transformed with the *35S* overexpression construct. NT_{seg} plants were used as controls.

Relative transcript levels were calculated using the $2^{-\Delta\Delta Ct}$ method and are shown relative to the corresponding non-transgenic control. Data were analyzed using one-tailed *t*-test (n = 3) comparing transgenic lines to non-transgenic controls, and are presented as mean ± SE.

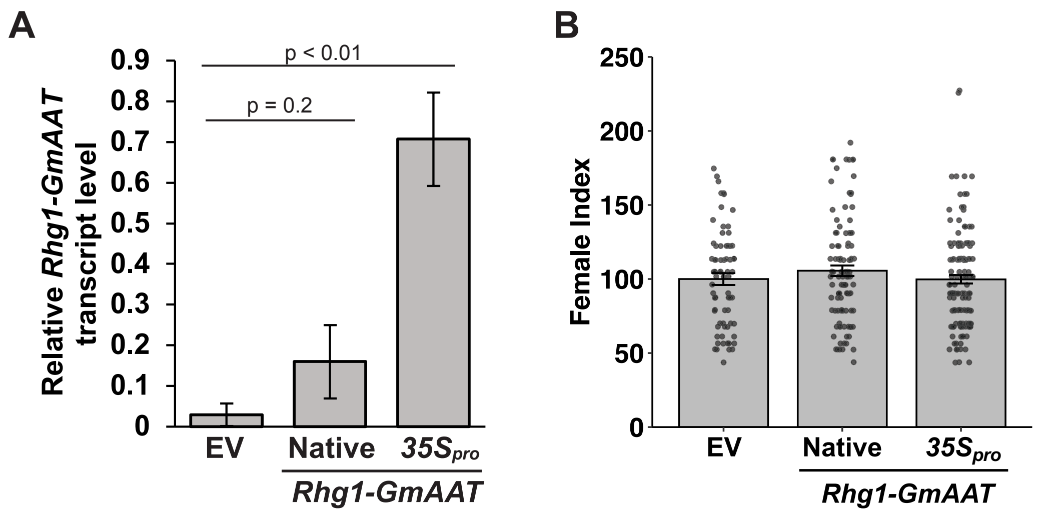

Supplemental Figure S5. Overexpression of *Rhg1-GmAAT* in Arabidopsis does not enhance resistance against BCN.

(A) Relative transcript abundance of *Rhg1-GmAAT* measured by RT-qPCR and normalized to *AtEF1a* in stable transgenic Arabidopsis lines transformed with an empty vector (EV), a native *Rhg1-GmAAT* construct (native promoter, UTRs, exons, introns and terminator) or a *35S promoter:Rhg1-GmAAT CDS:Nos terminator* overexpression construct. Data were analyzed using one-tailed *t*-test and are presented as mean ± SE (EV, n = 2; each transgenic line, n = 4).

(B) Transgenic Arabidopsis lines described above were tested against BCN. Female index was calculated relative to EV. ANOVA did not show significant differences. Data are presented as mean ± SE.

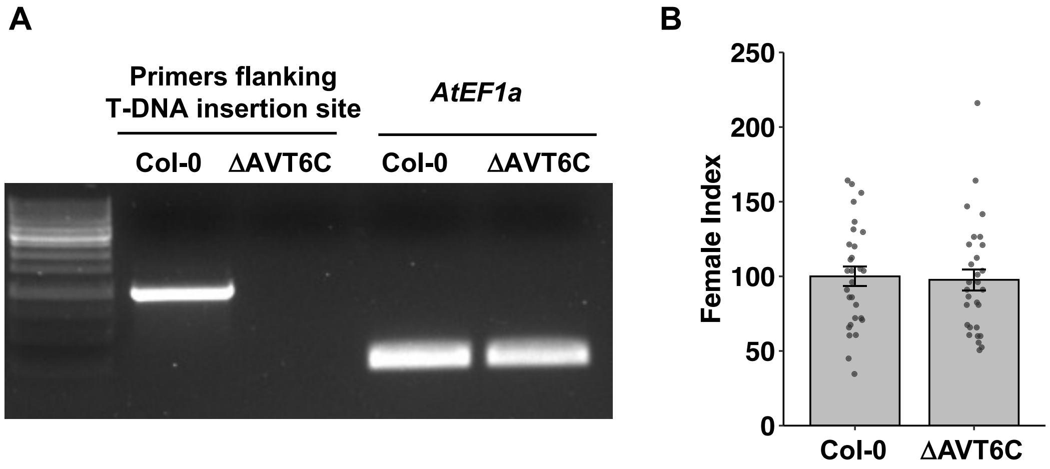

Supplemental Figure S6. Knocking out *AVT6C* in Arabidopsis does not impact BCN resistance.
(A) PCR genotyping of the *AVT6C* T-DNA insertion Arabidopsis line CS864997 (ΔAVT6C) obtained from Arabidopsis Biological Resource Center (ABRC). PCR was performed using primers flanking the T-DNA insertion site in the *AVT6C* gene, and genomic DNA extracted from Col-0 or ΔAVT6C plants as templates. A fragment of *AtEF1α* was amplified from the same genomic DNA templates as a control.
(B) The ΔAVT6C line was tested for resistance to BCN. Female index was calculated relative to Col-0. Data are presented as mean ± SE. Each bar contains four independent biological replicates. Each dot corresponds to data from an individual plant. A two-way ANOVA showed no significant differences between genotypes or replicates.

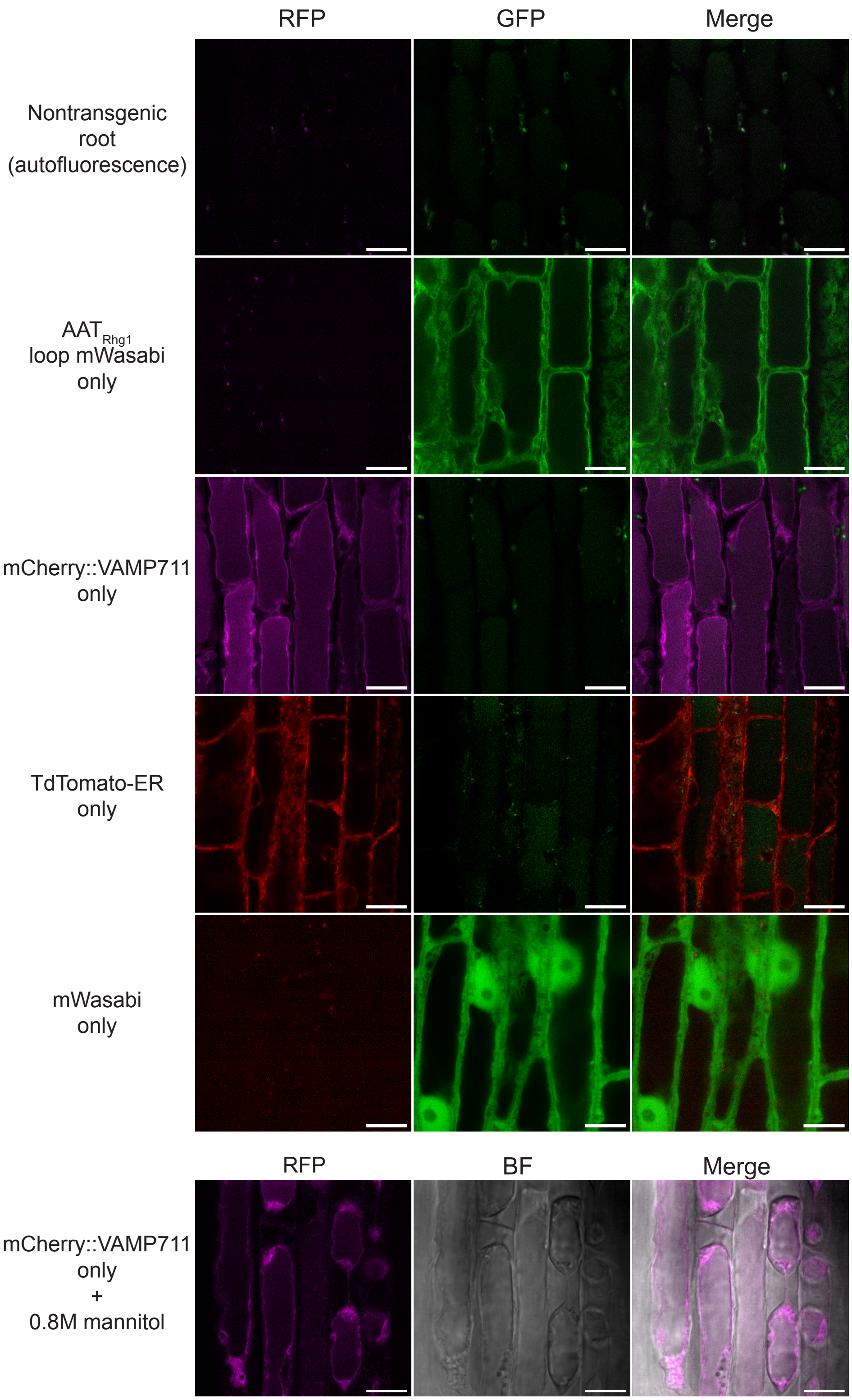

Supplemental Figure S7. Confocal imaging controls confirm channel specificity.

Representative confocal microscopy images of soybean roots showing, from top to bottom: non-transgenic roots (signal represents autofluorescence), AAT_{Rhg1} loop mWasabi only, mCherry::VAMP711 only, TdTomato-ER only, soluble mWasabi only, and mCherry::VAMP711 only treated with 0.8M mannitol for plasmolysis. No signal bleed-through was detected between RFP and GFP channels. Scale bars = 10 µm.

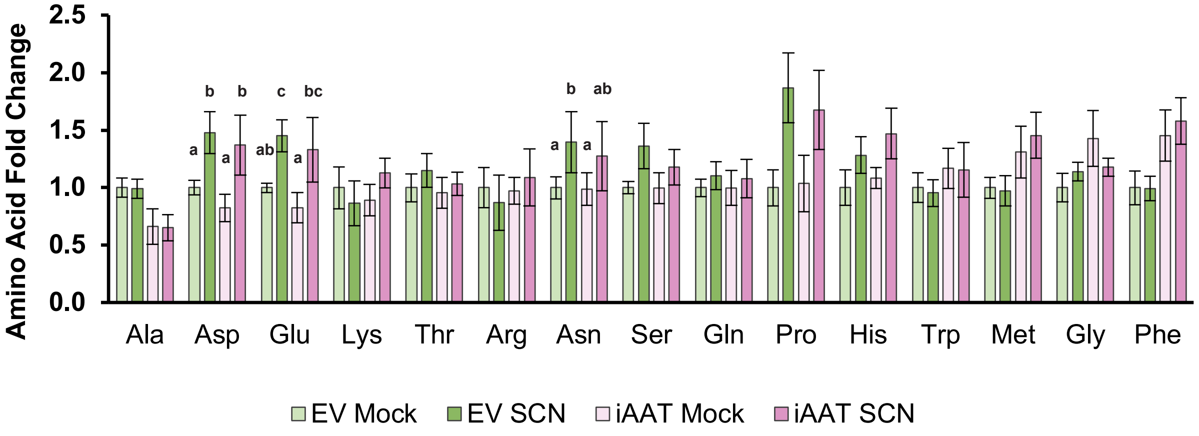

Supplemental Figure S8. Additional amino acid measurements in EV and *Rhg1-GmAAT*-silenced roots.

Quantification of amino acid levels in IL3025N (*rhg1-b*) empty vector (EV) or *Rhg1-GmAAT* RNAi-silenced (iAAT) transgenic soybean roots, either mock-treated or inoculated with SCN (HG 0) at 3 days post-inoculation (dpi). Amino acid levels were normalized as fold changes relative to EV-Mock. Data for each amino acid were analyzed using a linear model including group and replicate as fixed effects. Within each amino acid, bars sharing the same letter are not significantly different. Amino acids without letter annotations showed no significant differences. Data are presented as mean ± SE (n = 6, except Ala n = 3).

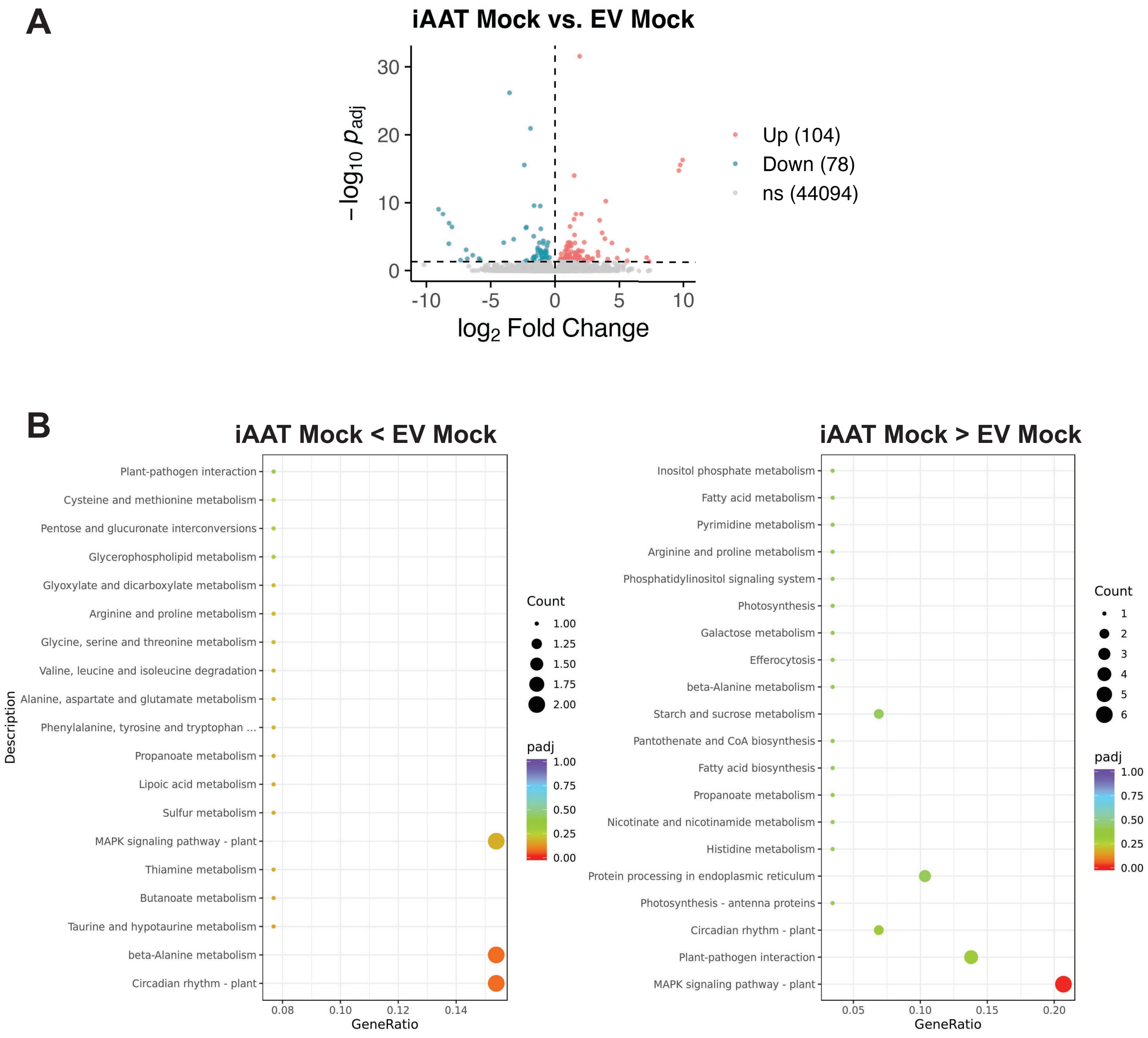

Supplemental Figure S9. Differential gene expression between iAAT and EV under mock conditions.

(A) Volcano plot showing differentially expressed genes (DEGs) between the mock-inoculated EV and iAAT roots. Each point represents a single gene. Genes with an adjusted *p*-value (p_{adj}) < 0.05 are highlighted.

(B) KEGG pathway enrichment analysis of DEGs identified in (A). Dot plots show the top 20 enriched KEGG pathways.

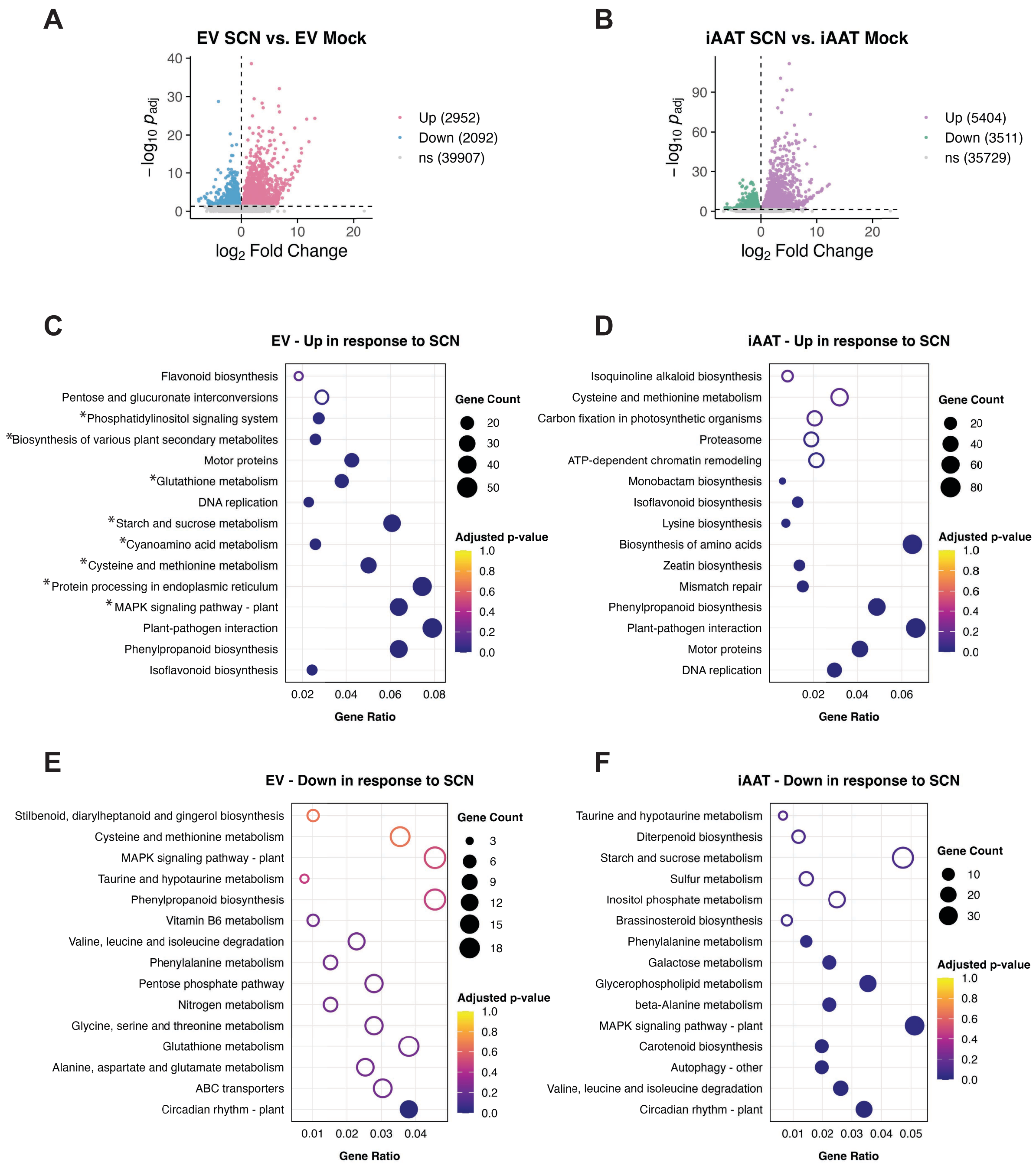

Supplemental Figure S10. Differential gene expression in EV and iAAT roots in response to SCN.
(A, B) Volcano plot showing differentially expressed genes (DEGs) between mock and SCN-inoculated conditions at 3dpi in the EV (A), or iAAT (B) roots. Each point represents a single gene. Genes with an adjusted *p*-value (p_{adj}) < 0.05 are highlighted. **(C-F)** KEGG pathway enrichment analysis of DEGs identified in (A) and (B). Dot plots show the top 15 enriched KEGG pathways based on upregulated genes in EV (C),

upregulated genes in iAAT (D), downregulated genes in EV (E), and downregulated genes in iAAT (F) in response to SCN. Dot size reflects the number of DEGs associated with each pathway, and dot color represents enrichment significance. Filled circles indicate significantly enriched pathways ($p_{adj} < 0.05$), while empty circles represent non-significant enrichment. KEGG pathways significantly enriched among SCN-upregulated genes in *rhg1-b* EV roots but not in iAAT roots are marked by asterisks in (C).

Abundance (FPKM) EV		Abundance (FPKM) iAAT		Gene	Abundance Difference (p_{adj}) SCN vs. mock		Abundance Difference (p_{adj}) iAAT vs. EV		DEG in over-represented MAPK signaling KEGG
mock	SCN	mock	SCN		EV	iAAT	mock	SCN	
11.1	10.4	11.7	9.2	*GLYMA_03G245000*	0.6451	0.0486	0.9999	0.7665	5'-3' exoribonuclease 4
15.9	16.3	17.6	13.3	*GLYMA_19G242400*	0.8839	0.033	0.9999	0.4143	5'-3' exoribonuclease 4
30.0	24.3	30.4	22.6	*GLYMA_04G017500*	0.1759	0.0264	0.9999	0.9128	catalase
64.4	44.7	67.7	41.5	*GLYMA_14G223500*	0.0509	0	0.9999	0.9106	catalase
40.1	31.9	45.2	28.5	*GLYMA_06G017900*	0.4203	0.0251	0.9999	0.8712	catalase
5.3	3.8	4.7	3.5	*GLYMA_03G086000*	0.2519	0.0263	0.9999	0.915	copper-transporting ATPase HMA4
16.4	21.6	24.5	14.8	*GLYMA_13G166200*	0.0908	0.0225	0.4631	0.2779	EIN3-binding F-box protein 1
40.4	30.9	48.2	25.8	*GLYMA_04G066900*	0.5174	0.0144	0.9999	0.8745	EIN3-binding F-box protein 1
17.7	19.6	23.6	12.4	*GLYMA_17G113900*	0.8116	0	0.9999	0.1987	EIN3-binding F-box protein 1
39.4	33.0	68.8	32.6	*GLYMA_17G211000*	0.4888	0.0001	0.1935	0.9888	EIN3-binding F-box protein 1
27.6	23.6	43.3	27.1	*GLYMA_14G116800*	0.6602	0.0191	0.6401	0.8544	EIN3-binding F-box protein 1
91.9	75.3	94.5	75.8	*GLYMA_02G274600*	0.1536	0.0036	0.9999	0.9962	ETHYLENE INSENSITIVE 3-like 1 protein
17.4	12.7	16.4	10.3	*GLYMA_13G342500*	0.2112	0.0031	0.9999	0.6183	ETHYLENE INSENSITIVE 3-like 3 protein
15.8	10.7	14.9	10.1	*GLYMA_15G031800*	0.0592	0.0002	0.9999	0.9363	ETHYLENE INSENSITIVE 3-like 3 protein
6.2	9.3	18.0	9.5	*GLYMA_20G202200*	0.2637	0.0238	0.0354	0.9888	ethylene receptor 2
33.1	25.2	31.8	22.0	*GLYMA_13G145100*	0.092	0.0386	0.9999	0.8113	ethylene-insensitive protein 2
1.6	1.9	12.1	3.1	*GLYMA_13G123100*	0.8095	0.0123	0.0168	0.6594	ethylene-responsive transcription factor 1-like protein
11.0	9.2	12.3	9.0	*GLYMA_10G167300*	0.4309	0.0407	0.9999	0.9795	GAF/HisKA/CheY-like REC domain-containing protein
17.1	13.3	16.8	13.4	*GLYMA_08G115200*	0.0735	0.0278	0.9999	0.9915	mitogen-activated protein kinase 7
13.5	11.8	12.0	9.7	*GLYMA_20G161500*	0.1335	0.0247	0.9999	0.2158	mitogen-activated protein kinase kinase kinase YODA
50.7	37.6	65.9	37.4	*GLYMA_13G076700*	0.1631	0	0.8172	0.9947	protein ETHYLENE INSENSITIVE 3
77.4	52.9	98.8	59.7	*GLYMA_20G051500*	0.0702	0	0.9709	0.8265	protein ETHYLENE INSENSITIVE 3
40.4	41.5	45.8	28.7	*GLYMA_04G205400*	0.9698	0.0306	0.9999	0.2555	protein kinase
34.2	43.9	50.1	34.9	*GLYMA_08G005100*	0.5652	0.0132	0.4379	0.8083	protein kinase 3
101.9	91.9	105.1	65.1	*GLYMA_14G066400*	0.7687	0.0199	0.9999	0.5167	protein phosphatase 2C 37
57.8	50.4	68.5	43.5	*GLYMA_17G052500*	0.5465	0.0001	0.9999	0.7555	protein phosphatase 2C 56
57.6	47.0	64.3	39.2	*GLYMA_13G106800*	0.4991	0.0063	0.9999	0.777	protein phosphatase 2C 77
41.8	35.0	64.1	30.8	*GLYMA_06G068400*	0.5953	0.0014	0.4701	0.9041	putative EIN3-binding F-box protein
10.1	8.5	11.2	7.7	*GLYMA_10G066000*	0.6399	0.0461	0.9999	0.8931	serine/threonine-protein kinase CTR1
9.3	8.4	8.5	6.2	*GLYMA_07G178600*	0.6985	0.0477	0.9999	0.454	serine/threonine-protein kinase SRK2E
29.2	22.2	30.4	23.6	*GLYMA_11G058800*	0.1204	0.0191	0.9999	0.927	serine/threonine-protein kinase SRK2I
16.9	12.6	14.5	10.5	*GLYMA_11G072900*	0.0903	0.0378	0.9999	0.7147	uncharacterized LOC100527909

Additional DEG in "Unique Down in iAAT"

EV mock	EV SCN	iAAT mock	iAAT SCN	Gene	SCN vs. mock EV	SCN vs. mock iAAT	iAAT vs. EV mock	iAAT vs. EV SCN	Additional DEG in "Unique Down in iAAT"
81.4	33.7	60.0	20.6	*GLYMA_07G264200*	0.074	0	0.9999	0.6406	1-aminocyclopropane-1-carboxylate oxidase
11.5	0.7	14.0	2.5	*GLYMA_08G169100*	0.0743	0.0486	0.9999	0.6078	1-aminocyclopropane-1-carboxylate oxidase homolog 11-like
37.3	18.7	44.4	23.3	*GLYMA_08G351866*	0.3567	0.0461	0.9999	0.8431	1-aminocyclopropane-1-carboxylate oxidase homolog 12
34.6	27.5	41.1	20.0	*GLYMA_07G168500*	0.4335	0.004	0.9999	0.4832	1-aminocyclopropane-1-carboxylate oxidase homolog 4
10.4	7.6	13.1	7.6	*GLYMA_07G021000*	0.2581	0.0233	0.9999	0.9978	AP2-like ethylene-responsive transcription factor At1g16060
37.4	25.6	43.2	21.5	*GLYMA_06G042100*	0.4663	0.0116	0.9999	0.8494	dehydration-responsive element binding protein 2
1.6	1.3	1.5	0.6	*GLYMA_09G052900*	0.8939	0.035	0.9999	0.7194	DNA-binding domain-containing protein
26.6	22.8	27.6	17.5	*GLYMA_10G058300*	0.4531	0.0004	0.9999	0.515	ethylene-insensitive protein 2
10.3	6.9	12.6	6.0	*GLYMA_11G167200*	0.4593	0.0001	0.9999	0.9124	ethylene-overproduction protein 1
5.2	3.0	4.7	2.9	*GLYMA_18G059700*	0.136	0.0002	0.9999	0.9428	ethylene-overproduction protein 1
78.6	64.2	144.0	80.8	*GLYMA_13G236500*	0.4711	0.0219	0.3586	0.7022	ethylene-responsive element binding factor 4
15.0	7.8	14.8	9.5	*GLYMA_08G137600*	0.0652	0.0165	0.9999	0.7601	ethylene-responsive transcription factor
42.6	43.3	73.0	36.3	*GLYMA_13G274100*	0.9752	0	0.5714	0.7325	ethylene-responsive transcription factor 4
14.6	10.9	15.9	10.3	*GLYMA_11G239200*	0.241	0.0078	0.9999	0.9585	ethylene-responsive transcription factor ERF060-like
0.1	1.1	6.5	1.4	*GLYMA_10G036200*	0.0315	0.0307	0.001	0.9245	ethylene-responsive transcription factor ERF096
2.4	3.7	10.7	2.2	*GLYMA_13G122500*	0.5031	0.001	0.033	0.7523	ethylene-responsive transcription factor ERF096
4.3	3.0	3.4	0.9	*GLYMA_10G223200*	0.7097	0.0022	0.9999	0.1836	ethylene-responsive transcription factor ERF110
1.1	0.2	2.9	0.5	*GLYMA_13G060600*	0.0552	0.0308	0.76	0.7739	ethylene-responsive transcription factor ERN2
15.7	10.3	21.6	7.3	*GLYMA_04G041200*	0.3871	0	0.9999	0.683	ethylene-responsive transcription factor RAP2-1
23.0	18.3	25.4	10.0	*GLYMA_12G073300*	0.675	0.0028	0.9999	0.2329	ethylene-responsive transcription factor RAP2-7
7.3	5.7	6.9	4.7	*GLYMA_09G177600*	0.2757	0.0405	0.9999	0.7396	probable serine/threonine-protein kinase SIS8
13.6	12.8	21.6	7.8	*GLYMA_14G182700*	0.8894	0.0005	0.911	0.3217	serine/threonine-protein kinase CTR1
14.1	11.8	14.2	11.0	*GLYMA_20G229500*	0.1194	0.0191	0.9999	0.8317	serine/threonine-protein kinase EDR1
15.0	14.7	25.2	15.7	*GLYMA_02G066200*	0.967	0.0364	0.8102	0.9456	uncharacterized LOC100796480

FPKM: 0 — 72 — 144

p_{adj}: 0.001 — 0.3 — 1.0

Supplemental Figure S11. Genes uniquely downregulated in iAAT in response to SCN that are associated with MAPK signaling and ethylene responses.
Top: Transcripts significantly downregulated at SCN infection sites in *Rhg1-GmAAT*-silenced roots (iAAT) but not in SCN-resistant *rhg1-b* (EV) roots, for over-represented KEGG group “MAPK signaling pathway – plant”. Bottom: Additional genes uniquely downregulated in iAAT but not in EV roots in response to SCN, that carry ethylene-associated annotation but are not placed by KEGG into the above group.

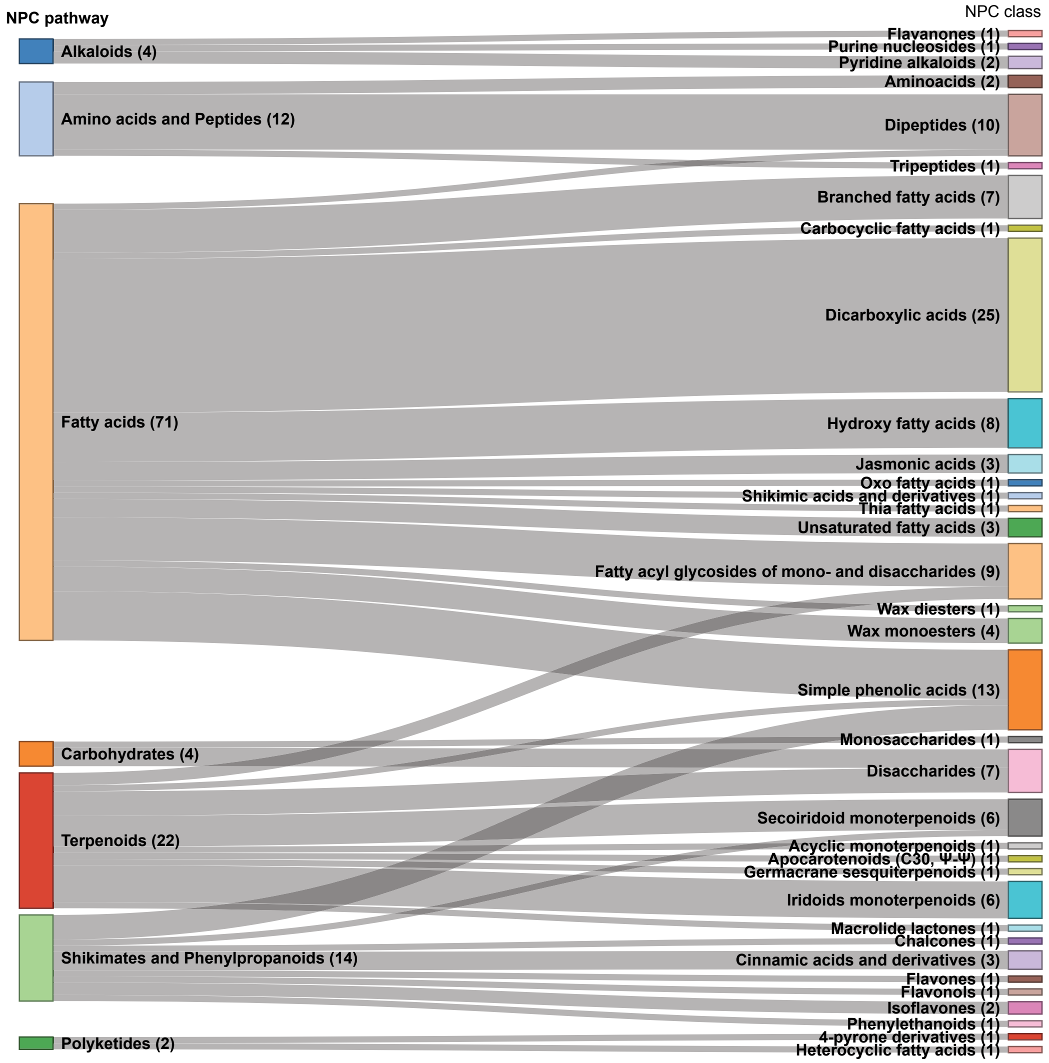

Supplemental Figure S12. Composition of differentially abundant annotated metabolite features detected in RP-negative mode.
Differentially abundant metabolite features (fold change > 2 or < 0.5; *t*-test $p < 0.05$, as defined in Fig. 7B and 7C) with available NPC annotations were grouped by NPC pathways and classes. Bars represent the distribution of these annotated metabolites.

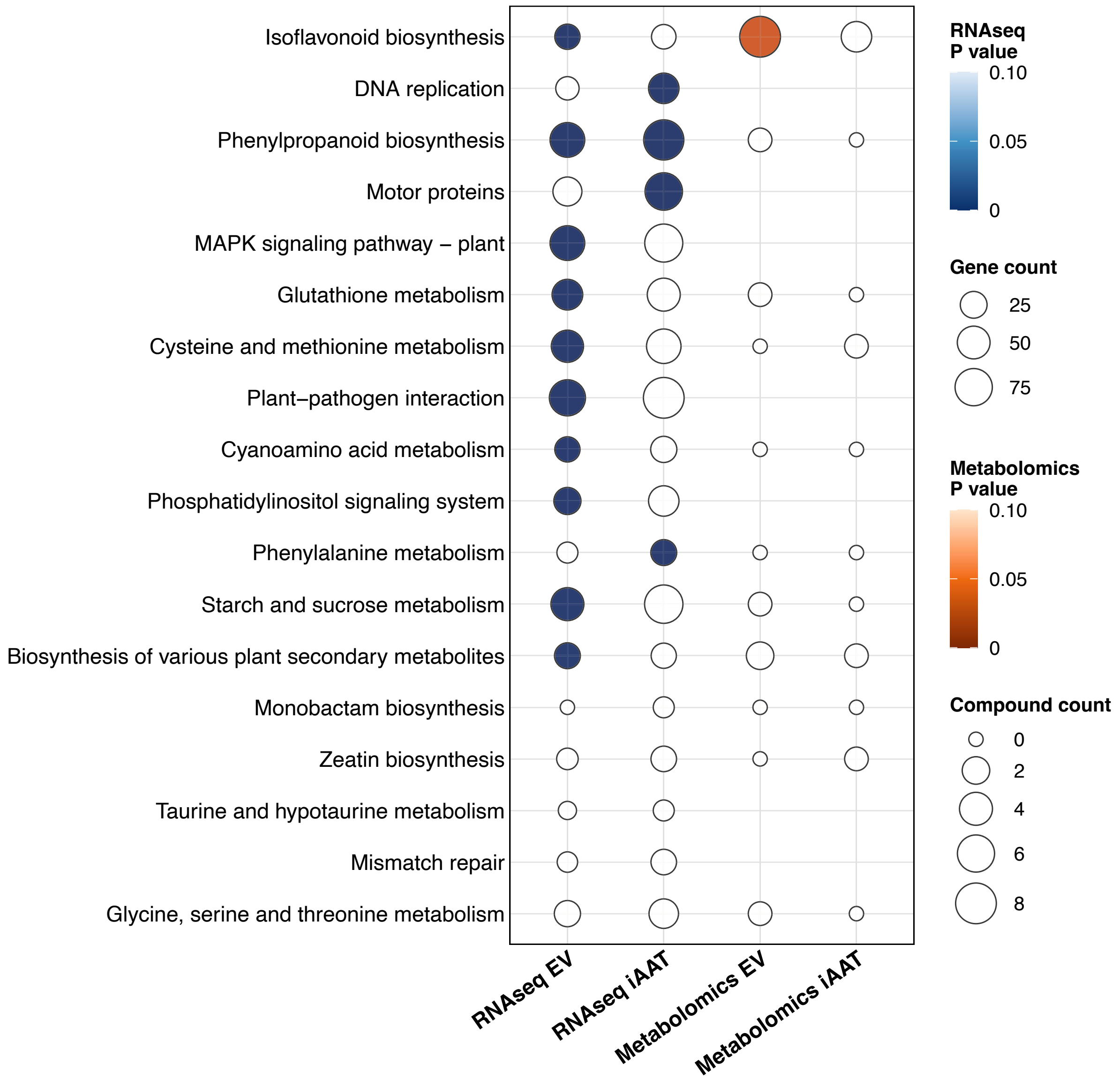

Supplemental Figure S13. KEGG enrichment comparison of SCN-responsive genes and metabolites in EV and iAAT roots.
Dot plot shows RNA-seq KEGG enrichment and metabolomics KEGG enrichment. RNA-seq enrichment was based on SCN-responsive genes (p_{adj} < 0.05, both up and down), and metabolomics enrichment was based on differentially abundant metabolite features (p < 0.05) mapped and deduplicated to KEGG compounds. Dot size represents the number of genes or KEGG compounds contributing to each pathway. Filled circles indicate significant enrichment, with color showing the enrichment *p*-value; unfilled circles indicate non-significant enrichment. RNA-seq and metabolomics *p*-values are shown using separate color scales.

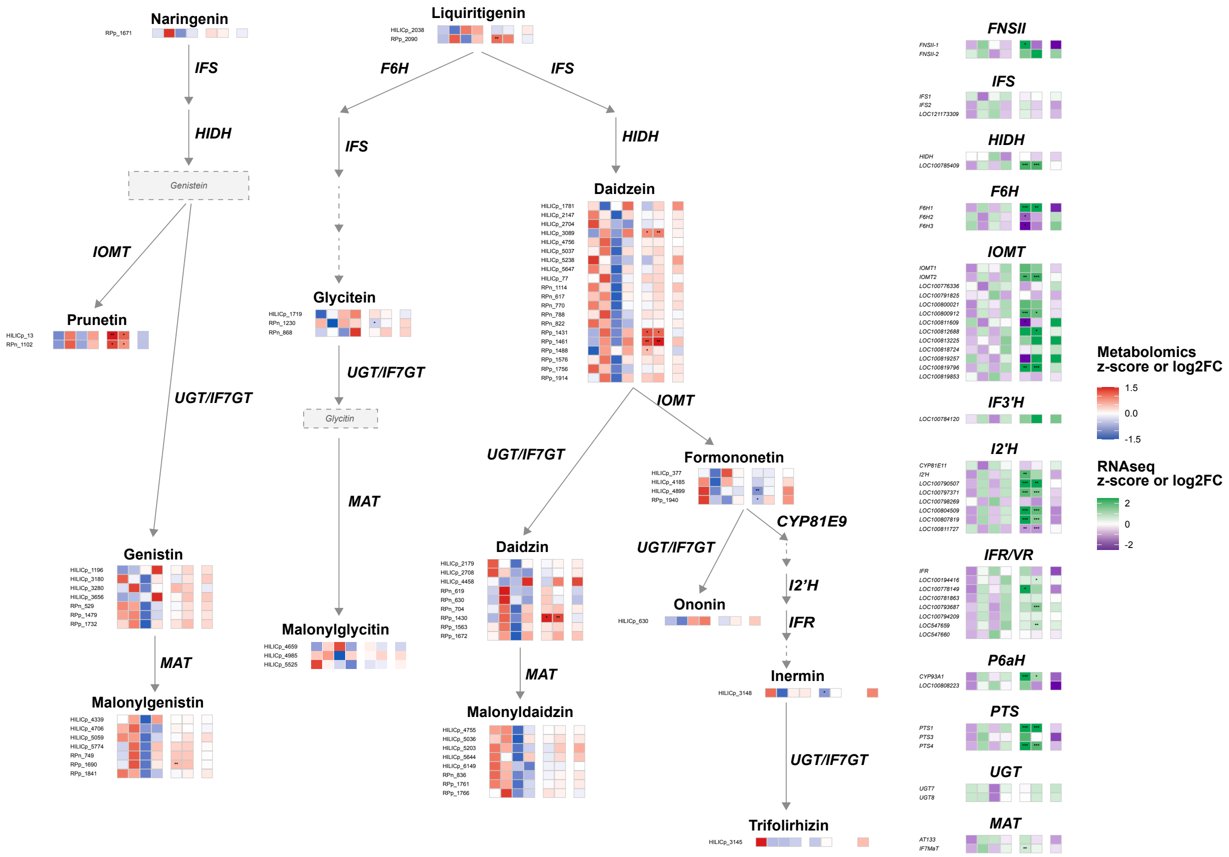

Supplemental Figure S14. Isoflavonoid biosynthesis pathway with transcriptomic and metabolomic changes in response to SCN.
Simplified isoflavonoid biosynthesis pathway based on KEGG gmx00943, showing detected KEGG-mapped metabolite features and KEGG-associated genes in EV and iAAT roots under mock or SCN treatment at 3 dpi. Right-side panels show the transcriptomic heat maps of genes in the KEGG isoflavonoid biosynthesis pathway, including branches not fully represented in the simplified metabolite pathway on the left. Dashed metabolite boxes indicate pathway intermediates included for pathway context but not detected in the untargeted metabolomics dataset, and dashed arrows indicate condensed or unresolved KEGG steps. Heatmap tile columns are EV mock, EV SCN, iAAT mock, iAAT SCN; EV SCN/mock, iAAT SCN/mock; and the difference between iAAT SCN/mock and EV SCN/mock. Group tiles show row z-scores, and response tiles show log2 fold-change values. Asterisks indicate significant SCN-responsive changes: **p* < 0.05, ***p* < 0.01, and ****p* < 0.001. RNA-seq significance is based on adjusted *p* values, and metabolomics significance is based on raw *p* values.

Extended Materials and Methods Descriptions

For Du et al. "AAT_{Rhg1} is a tonoplast protein that alters amino acid, metabolic and defense responses and nematode resistance"

Plasmid constructs

The *Rhg1-GmAAT* silencing construct was described in (Du *et al*., 2025). In brief, the hairpin RNA construct was generated using a 292 bp DNA fragment spanning the junction between *Rhg1-GmAAT* first and second exons, followed by a 523bp GUS fragment as a spacer and then the same 292bp fragment in reverse complement orientation, cloned into pCambia2300 plasmid under the control of a *GmUbi* promoter (Hernandez-Garcia *et al.*, 2010) and an *Rbcs-E9* terminator. A spectinomycin resistance marker with a chloroplast targeting signal driven by a double Cauliflower mosaic virus (CaMV) *35S* promoter and nopaline synthase (*nos*) terminator was used for selecting whole transgenic soybeans (Martinell *et al.*, 2013).

An artificial RNAi-escaping *Rhg1-GmAAT* coding sequence (CDS) was created by designing synonymous codon substitutions based on soybean codon usage table. A DNA fragment containing the designed RNAi-escaping *Rhg1-GmAAT* CDS with BbsI cutting sites flanking the CDS was synthesized at Twist Bioscience (South San Francisco, CA, USA) and cloned into a Golden Gate level 0 and then level 1 vector under control of a *GmUbi* promoter and *nos* terminator. The *Rhg1-GmAAT* expression module together with *2xCaMV* $35S_{pro}$*:TMV omega enhancer:ZsGreen-KDEL:*NOS_{ter} were cloned into binary vector pAGM4673, with the *ZsGreen* module next to the left border of the T-DNA as the screenable marker for transgenic soybean roots. Single

amino acid mutation *Rhg1-GmAAT* alleles were generated by site-directed mutagenesis by PCR with overlapping oligonucleotide primers, followed by *DpnI* digestion of template DNA and transformation into *E. coli*.

To investigate the effect of elevated *Rhg1-GmAAT* transcript levels on SCN resistance, two T-DNA cassettes were constructed and inserted separately into binary vector pAGM4673, each along with a spectinomycin resistance marker gene expression cassette for selection of transgenic plants. The native *Rhg1-GmAAT* construct was built from the Williams82 *Glyma.18G022400* genomic sequence, including the full 1,546 bp native promoter spanning the region upstream of the 5' untranslated region (UTR) to the transcription start of divergently transcribed *Glyma.18G022500*, and also the complete coding sequence including introns and UTRs followed by the 440 bp downstream region serving as the native terminator. The overexpression construct consisted of the *Rhg1-GmAAT* coding sequence (exons only) controlled by a double CaMV *35S* promoter with a TMV *omega* enhancer, followed by the *nos* terminator.

For microscopy, genes encoding the fluorescent proteins mCherry, TdTomato with C-terminal KDEL endoplasmic reticulum (ER) retention signal, and mWasabi were obtained as Golden Gate level 0 plasmids. Subsequent cloning was done using the Golden Gate Assembly MoClo Tool Kit (Weber *et al.*, 2011). Green fluorescent protein mWasabi was tagged to the N or C terminus of AAT_{Rhg1} in Golden Gate level 0 to level 1 reactions. The loop-tagged AAT_{Rhg1} was created through Gibson Assembly. PCR reactions were performed to amplify *Rhg1-GmAAT* and *mWasabi* CDS with compatible overhangs added for Gibson Assembly. *VAMP711* was cloned from Arabidopsis Biological Resource Center (ABRC) CD3-781601, and inserted into Golden Gate level 0

vector through BbsI overhangs added by PCR. The above genes were cloned into Golden Gate level 1 vectors for expression under the control of double CaMV *35S* promoter with TMV *omega* translational enhancer and *nos* terminator. The level 1 modules were assembled into the Golden Gate level 2 binary vector to generate constructs for co-expression in soybean roots.

A list of the oligonucleotide primers used is provided as Supplemental Table S1.

Transgenic plant materials

IL3025N (synonyms: Illini 3025N, LD11-2170; *rhg1-b* SCN resistance haplotype) and IL3849N (synonyms: Illini 3849N, LD07-3395bf; *rhg1-a* + *Rhg4* SCN resistance haplotypes) were developed by Brian Diers, University of Illinois at Urbana-Champaign, and are available through Baird Seed Co. (Williamsfield, Illinois). The pedigree for IL3025N is Syngenta_03JR313108 x LD05-3171. IL3849N is an SCN-resistant reselection from LD07-3395 whose pedigree is Syngenta_WW115926 x LD00-2817. Whole transgenic *Rhg1-GmAAT*-silenced soybean plants were generated from IL3025N at Wisconsin Crop Innovation Center (WCIC) as described in Du *et al.*, 2025. Transgenic lines were selected using the spectinomycin resistance marker (Martinell *et al.*, 2013) and T-DNA copy numbers were determined by qPCR using genomic DNA (more details in “DNA extraction and copy number test” section). Transgene expression or target gene silencing were verified by RT-qPCR. For the *Rhg1-GmAAT*-silenced RNAi and corresponding EV lines, stable homozygous transgenic lines were selected, and 4th generation (T4) transgenic plants were used in SCN assays. For *Rhg1-GmAAT* overexpression lines generated in Williams 82 (Wm82) background, homozygous T2

plants were used in SCN assays. For *Rhg1-GmAAT* overexpression lines generated in IL3025N and IL3849N, T1 plants were used in SCN assays. Because the T1 siblings from the same event segregated and are a mixture of homozygotes, hemizygotes and non-transgenic, T-DNA copy number was tested for each plant used in SCN assays.

Composite soybean plants with transgenic roots were generated from soybean seedling hypocotyls using *Agrobacterium rhizogenes* strain K599 following the methods of Estrada-Navarrete & Alvarado-Affantranger (2007) and Fan *et al*. (2020). Detached transgenic soybean roots were produced from soybean cotyledons using the method described in Cook *et al*. (2012).

Transgenic Arabidopsis lines expressing *Rhg1-GmAAT* were generated using the floral dip method (Bent, 2006) and homozygous transgenic lines were identified using the spectinomycin resistance gene described above (Martinell *et al*., 2013). *AtAVT6C* knockout line CS864997 was ordered from ABRC (Arabidopsis Biological Resource Center, Ohio State University, Columbus OH).

DNA extraction and copy number test

DNA was extracted from young soybean leaf tissue after flash-freezing in liquid nitrogen, using the CTAB/chloroform method. Transgene copy number was tested by qPCR, measuring the relative level of the spectinomycin resistance gene on T-DNA relative to *Glyma.18G022800*, which is known to only have one copy per haploid genome.

Confocal microscopy

Confocal images were acquired using a Zeiss LSM 980 with 40x water immersion objective lens. TdTomato or mCherry signal was acquired with 561 nm laser excitation and 573 to 628 nm emission, while mWasabi signal was acquired with 488 nm laser excitation and 500 to 553 nm emission with beam splitter MBS 488/561 and laser intensity 2.0%.

SCN propagation and resistance assay

SCN populations were initially collected in the field and propagated in the laboratory on soybean plants grown in a 1:1 soil sand mixture. The HG 0 population used in experiments was originally collected in Illinois. Two HG 2.5.7 populations were sourced from Minnesota (HG 2.5.7 MN) or Missouri (HG 2.5.7 MO). To maintain or enhance virulence, these SCN populations were propagated on different soybean varieties: HG 0 on the susceptible variety Wm82 and HG 2.5.7 on *rhg1-b* variety IL3025N. Propagation pots were grown for approximately two months before harvesting. SCN cysts and eggs were extracted using the method described in Cook *et al.*, 2012. Eggs were hatched in 4 mM pH 5.6 $ZnCl_2$ solution.

SCN resistance assays were conducted in a controlled temperature growth room set for 16-hour light (~800 μmol/m^2/sec) at 28°C. Each transgenic plant was planted individually in 6cm pots containing a 1:1 soil-sand mixture and inoculated with nematodes. Whole transgenic plants were each inoculated with ~1000 SCN eggs and incubated for 35 days, while composite plants were each inoculated with ~750 freshly hatched SCN J2s and incubated for 30 days. Felt strips were placed at the bottom of

each pot to wick water. Soybean plants with different genotypes within a single experiment were anonymized with random numbering and arranged in a completely randomized design. After the incubation period, cysts were extracted from soil by water flotation, then collected on nested U.S. Standard No. 25 (710 μm opening) and No. 80 (180 μm opening) sieves, followed by separation using 68.1% (w/v) sucrose solution. Cysts harvested from each plant were counted using a stereo dissecting microscope.

A sugar beet cyst nematode (*Heterodera schachtii*, BCN) population was obtained from Dr. Thomas J Baum and maintained on collard. BCN assays on Arabidopsis were conducted as described by Butler *et al*. (2019).

RNA extraction, RT-qPCR, and RNA-seq

Sampled plant tissues were flash-frozen in liquid nitrogen and stored at -80°C until RNA extraction. Frozen samples were mechanically ground with 2mm zirconium silicate bead and PowerLyzer™ 24 (MO BIO Laboratories, Inc., Carlsbad, CA, USA). RNA was extracted using the Direct-zol™ RNA MiniPrep Plus Kit (Zymo Research, Irvine, CA, USA) according to the manufacturer's protocol. To obtain high-quality RNA for RNA-seq, an additional chloroform phase separation step was included, adapted from the TRIzol™ Reagent user guide (Thermo Fisher Scientific, Waltham, MA, USA). Specifically, 1 mL of TRIzol™ was added to each sample, followed by the addition of 200 μL of chloroform. The tubes were then centrifuged at 12,000 x g for 15 minutes at 4°C. The aqueous phase was transferred to a new tube, mixed with an equal volume of 100% ethanol, and processed through the Direct-zol™ column. The extraction was then

completed following Direct-zol™ manufacturer's instructions. Complementary DNA (cDNA) was synthesized using FIREScript® RT cDNA synthesis MIX from Solis BioDyne (Tartu, Estonia). Quantitative reverse transcription polymerase chain reaction (RT-qPCR) was performed using HOT FIREPol® EvaGreen® qPCR Supermix from Solis BioDyne and CFX96 real-time PCR detection system (BioRad, Hercules, CA, USA).

For RNA-seq studies, transgenic soybean plants were germinated in vermiculite for 4 days before being transplanted to sand individually. Ten days after germination, each plant was inoculated with 1000 freshly hatched SCN (HG 0) J2s or the same volume of SCN-free hatching solution for the mock treatment. Three days after inoculation, root segments with visible lesions caused by nematode penetration were collected from SCN-inoculated plants by freezing in liquid nitrogen within one minute of excision, and root segments from corresponding regions on mock-inoculated plants were sampled. Four replicate samples were analyzed for each genotype-treatment. Each replicate was comprised of multiple root sections from one individual plant. RNA-seq and data analysis were performed by Novogene (Sacramento, CA, USA) using their Eukaryotic mRNA-seq service (Illumina NovaSeq platform, 150bp paired-end reads, ≥ 20 million read pairs per sample, read quality control, and gene expression quantification). The iAAT_Mock group had three replicates after one outlier was removed. Differential expression profiling primarily utilized Novogene platform for fpkm, log2 fold change and adjusted p value (p_{adj}) data, as well as KEGG and GO term functional group enrichment analysis.

Metabolite extraction and quantification

For metabolite extraction, seeds were initially germinated in vermiculite and transferred to sand four days post-germination. At 12 days post-germination, each plant was either inoculated with 1,000 SCN J2 juveniles or mock-treated. Three days post-inoculation (dpi), root infection zones under SCN (HG 0) infection or corresponding regions from mock-treated plants were collected. For each sample, root sections from three individual plants were pooled, flash-frozen in liquid nitrogen, and freeze-dried for 48 hours using the FreeZone® 4.5L Benchtop Freeze Dryer (Labconco, Kansas City, MO, USA).

Metabolites were extracted using a 2:1 mix of methanol and chloroform, with 50 µM of $^{13}C_6$-ring-labeled-L-phenylalanine as internal standard. Approximately 50 mg of freeze-dried root tissue were added to 800 µL of extraction solvent, then vortexed continuously at ambient temperature for 45 minutes. After spinning at 20,000 *g* for five min, the supernatant (~800 µL) was transferred to a clean tube and mixed with 600 µL of LC-MS grade water and 300 µL of chloroform by vortexing for ~one minute. Following a second centrifugation step at 20,000 *g* for five min, the top aqueous layer (~1 mL) was recovered and evaporated overnight in a SpeedVac at room temperature. Dried extracts were resuspended into 50 µL of 80% methanol by vortexing and sonication in a water bath for five min, followed by centrifugation at 20,000 *g* for five min. The final supernatant was collected and transferred to vials for LC-MS analysis.

For LC-MS analysis, a Vanquish Horizon Binary UHPLC (Thermo Scientific, Waltham, MA, USA) coupled to a Q Exactive Orbitrap mass spectrometer (Thermo Scientific, Waltham, MA, USA), was used. Amino acids were analyzed by zwitterionic hydrophilic interaction liquid chromatography (HILIC-Z) using an InfinityLab Poroshell

120 HILIC-Z column (2.7 µm particle size, 150 × 2.1 mm) (Agilent, Santa Clara, CA, USA) in a 22 min gradient of A (5 mM ammonium acetate, 0.2% acetic acid in water) in B (5 mM ammonium acetate, 0.2% acetic acid in 95% acetonitrile) at a flow rate of 0.45 mL/min: 0 to 1 min, isocratic 100% B; 1 to 7.5 min, 100% to 82% B; 7.5 to 11 min, 82 to 63% B; 11 to 14 min, 63% to 15% B; 14 to 16.5 min, isocratic 15% B; 16.5 to 17.5 min, 15% to 100% B; 17.5 to 22 min, isocratic 100% B. Total ion current (TIC). MS1 and MS2 data were collected between 1 and 16.5 minutes in full scan mode between *m*/*z* 70 to 1050 in positive ionization. MS/MS settings for HILIC-Z chromatography were described in El-Azaz & Maeda, 2024. Phenylpropanoids and other semi-polar metabolites were determined by reversed-phase (RP) chromatography using an Acquity UPLC HSS T3 column (1.8 µm particle size, 2.1 mm X 100 mm) (Waters, Milford, MA, USA) using two alternative gradients of A (0.1% formic acid in water) in B (0.1% formic acid in 90% acetonitrile) depending of the ionization mode (negative or positive, see below). The following 30 min gradient at 0.4 mL/min was used in negative ionization mode: 0 to 1 min, isocratic 1% B; 1 to 5 min, 1% to 10% B; 5 to 20 min, 10% to 25% B; 20 to 22 min, 25% to 99% B; 22 to 25 min, isocratic 99% B; 25 to 26 min, 100% to 1% B; 26 to 30 min, isocratic 1% B. For detection of phenylpropanoids and other semi-polar metabolites in positive ionization mode, the following 27 min gradient at 0.4 mL/min was used: 0 to 1 min, isocratic 1% B; 1 to 10 min, 1% to 10% B; 10 to 13 min, 10% to 25% B; 13 to 18 min, 25% to 99% B; 18 to 22 min, isocratic 99% B; 22 to 23.5 min, 99% to 1% B; 23.5 to 27 min, isocratic 1% B. MS1 and MS2 data were collected between 0.5 and 25 min (for the negative ionization method) and between 0.5 to 22 min (for the positive ionization method) using full scan mode between m/z 60 to 900. All other

MS/MS settings for RP chromatography were described in Du *et al*., 2025. Untargeted metabolite data processing and metabolite annotation were performed using MZmine v4.0 (Schmid *et al.*, 2023) and SIRIUS v5 (Dührkop *et al.*, 2019) as described in El-Azaz and Maeda, 2024. For targeted metabolite analysis, compound abundance was calculated based on the peak area of authentic standards and normalized to sample mass and internal standard recovery for each sample.

Association Analysis of Transcriptome and Metabolome Data

For metabolomics differential abundance analysis, raw peak areas from each LC-MS mode were compared between SCN-treated and mock-treated roots within each genotype. Two-sided Welch's t-test p-values were calculated and were adjusted using the Benjamini-Hochberg method, but metabolite features with raw $p < 0.05$ were used for the exploratory KEGG enrichment analysis.

Annotated metabolite features were mapped to KEGG compound IDs by name matching. When multiple LC-MS features mapped to the same KEGG compound, the feature with the smallest *p*-value was retained so that enrichment was performed at the compound level rather than the feature level. For metabolite KEGG enrichment, the background was defined as all KEGG-mapped compounds detected in the metabolomics dataset that were linked to at least one KEGG pathway. Compound-to-pathway relationships were obtained from KEGG, and pathways containing fewer than two background compounds were excluded. For each pathway, enrichment of significant compounds was tested using a hypergeometric test, followed by Benjamini-Hochberg correction across tested pathways.

For the RNA-seq-metabolomics KEGG comparison, RNA-seq KEGG enrichment results for EV and iAAT were combined with the metabolite compound-level KEGG enrichment results. RNA-seq significance was defined as pathway $p_{adj} < 0.05$, whereas metabolomics significance was defined as pathway raw $p < 0.05$.

The public web interface of the AI agent Biomni (https://biomni.stanford.edu) (Huang *et al.*, 2025) was utilized for an initial exploratory transcriptome-metabolome integration analysis to guide workflow development. Final analyses and figures were independently reproduced and verified by authors.

Statistical analyses

RNA-seq analyses are described separately. All other statistical analyses, including principal component analysis, were performed in R (version 4.4.2). Differences between two groups were evaluated using Student's *t*-test when data met assumptions of normality, or the Wilcoxon rank-sum test otherwise. For comparisons among more than two groups, one-way or two-way analysis of variance (ANOVA) followed by Tukey's HSD post hoc test was used. A *p*-value < 0.05 was considered statistically significant.
